# What Are the Sources of Unclassified Organosulfates in the Atmosphere?

**DOI:** 10.1021/acs.est.6c08394

**Published:** 2026-09-01

**Authors:** Donger Lai, Thomas Schaefer, Ru-Jin Huang, Jian Zhen Yu, Hartmut Herrmann, Man Nin Chan

**Affiliations:** † Department of Earth and Environmental Sciences, Faculty of Science, 26451 The Chinese University of Hong Kong, Hong Kong 999077, China; ‡ Atmospheric Chemistry Department (ACD), 28397 Leibniz Institute for Tropospheric Research (TROPOS), Leipzig 04318, Germany; § State Key Laboratory of Loess Science, Institute of Earth Environment, 53047 Chinese Academy of Sciences, Xi’an 710061, China; ∥ Institute of Global Environmental Change, Xi’an Jiaotong University, Xi’an 710049, China; ⊥ University of Chinese Academy of Sciences, Beijing 100049, China; # Division of Environment and Sustainability, The Hong Kong University of Science and Technology, Hong Kong 999077, China; ∇ Department of Chemistry, The Hong Kong University of Science and Technology, Hong Kong 999077, China; ○ School of Environmental Science and Engineering, Shandong University, Qingdao 266237, China; ◆ Shanghai Key Laboratory of Atmospheric Particle Pollution and Prevention, Department of Environmental Science & Engineering, Institute of Atmospheric Sciences, Fudan University, Shanghai 200433, China; ¶ The Institute of Environment, Energy, and Sustainability, The Chinese University of Hong Kong, Hong Kong 999077, China

**Keywords:** organosulfur compounds, organosulfates, transformation, inorganic sulfate, aerosol sulfur form

## Abstract

Organosulfates (OSs) are ubiquitous components of atmospheric aerosols, contributing up to 30% of total aerosol mass, yet their sources remain poorly constrained. Recently, we have compiled 366 ambient OS molecular formulas from previous field studies and found that only about 36% can be assigned to known precursors or formation mechanisms. These mechanisms primarily explain the production of freshly formed OSs via photochemical and/or condensed-phase reactions associated with different precursors. Consequently, 64% of observed OS formulas remain unclassified. Concurrently, laboratory studies have revealed that certain OSs can undergo efficient multiphase transformation through heterogeneous and aqueous-phase reactions, yielding higher-generation OS products. Although such studies are still limited, the identified transformation OS products can account for about 17% of all reported ambient OS formulas and, notably, explain an additional 5% of previously unclassified OS signals. These findings imply that a potential fraction of ambient OSs may not be freshly formed but rather secondary or higher-generation products arising from chemical transformation. Current field analyses and atmospheric models focus mostly on precursor-specific OS formation pathways, largely overlooking the role of OS transformation. We therefore propose that transformation of freshly formed OSs is an important and previously underrecognized source of unclassified ambient OSs.

## Graphical abstract

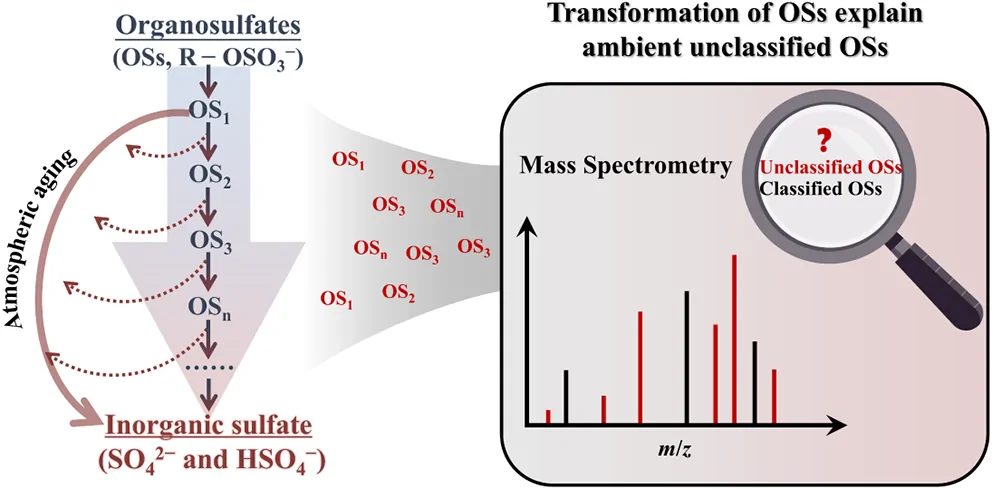

For many decades, atmospheric aerosol sulfur has often been viewed as existing primarily in inorganic forms (e.g., bisulfate (HSO_4_^–^) and sulfate (SO_4_^2–^)) because of its dominant role in regulating aerosol composition and physicochemical properties.^1,2^ This view has been considerably broadened during the last 20 years by extensive field observations showing that organosulfur compounds, particularly organosulfates (OSs, R–OSO_3_^–^), are also ubiquitous in ambient aerosols.^3–5^ OSs have been shown to affect aerosol physicochemical properties, including surface activity, hygroscopicity, viscosity, light absorption, and their role in new particle formation.^6–10^ Despite this progress, our understanding on the composition, molecular structures, formation mechanisms, and transformation pathways of OSs are still very limited. As shown in Figure 1a and b, we have recently compiled data sets of OSs identified in field studies and found a large and diverse set of total 366 OS molecular formulas (Table S1), of which only 130 (∼36%) can be linked to primary emissions or specific precursors using currently proposed formation mechanisms. These mechanisms primarily explain the production of freshly formed OSs via photochemical reactions and/or condensed-phase processes associated with different precursors, including the oxidation of various volatile organic compounds (VOCs) in the presence of sulfate particles, sulfate esterification reactions, nucleophilic substitution of alcohols by sulfuric acid, aqueous-phase sulfoxy radical reactions, heterogeneous reactions involving SO_2_, and gas-phase OS formation followed by gas-to-particle partitioning.^1,10–21^ Correspondingly, the majority of the observed OS molecular formulas (64%) remains unclassified and cannot be attributed to any known sources or established formation pathways (Figure 1b and c). This highlights the potential limitations of relying solely on fresh OS formation chemistry to account for the complexity of ambient OSs and raises a question: what are the sources of these ambient OSs, particularly the sizable unclassified fraction?

**Figure 1:**
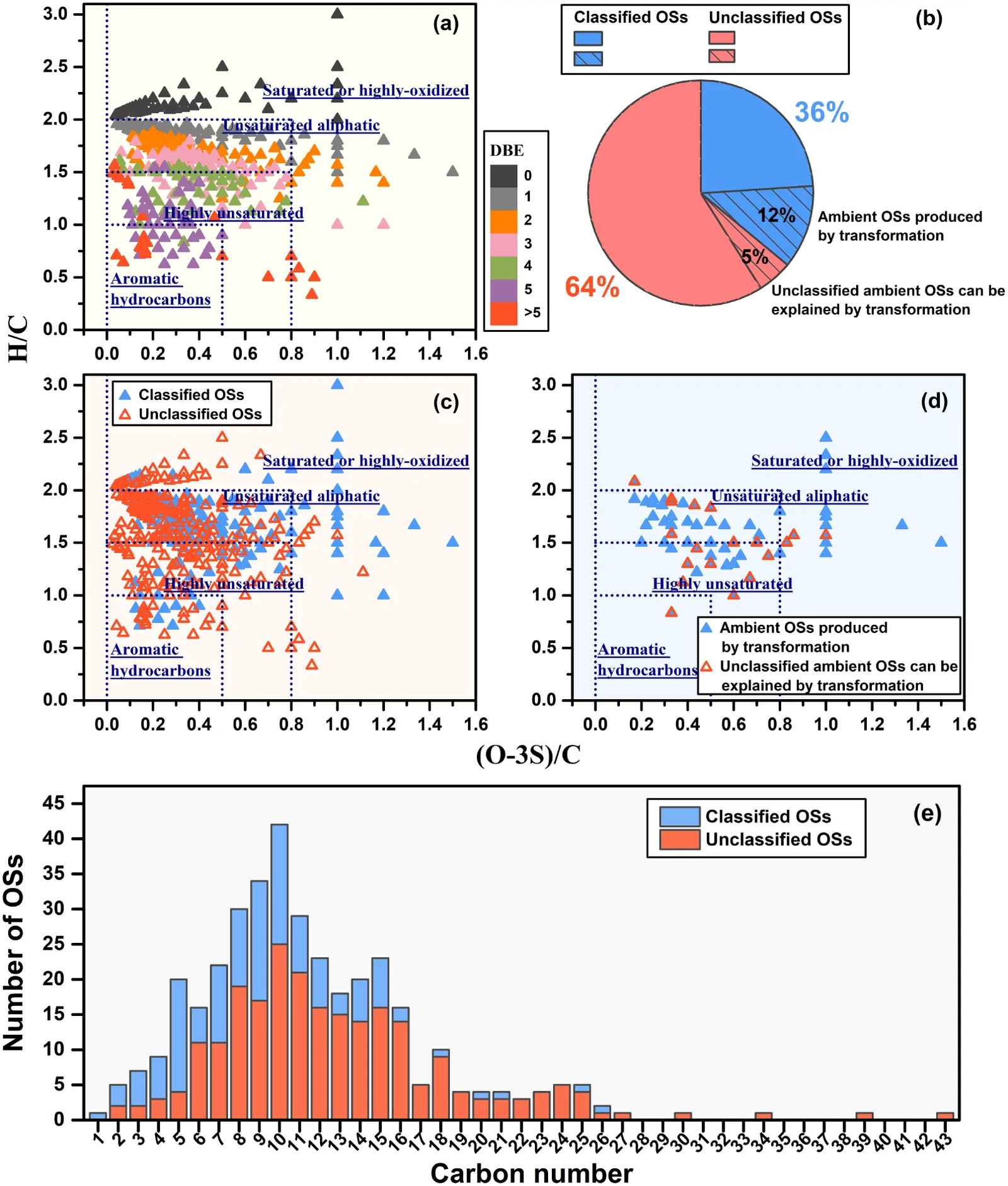
(a) Van Krevelen diagrams (H/C vs (O–3S)/C) for ambient OS molecular formulas compiled in Table S1, (O–3S)/C is used as a proxy for OS oxidation state. All detected OS formulas, colored by double bond equivalent (DBE) values. (b) Pie chart summarizing the percentages of classified vs unclassified OS formulas. (c) Same data set, with classified OSs (solid blue triangles) and unclassified formulas (open red triangles). (d) Same data set, highlighting OS formulas produced by transformation (solid blue triangles) and the unclassified ambient OS formulas can be explained by transformation (open red triangles). Dashed boxes denote approximate compositional domains (e.g., aromatic hydrocarbons, highly unsaturated, unsaturated aliphatic, and saturated/highly oxidized). (e) Distribution of classified and unclassified OSs by carbon number.

Table S1 compiles the molecular characteristics of ambient OSs. Figure 1a shows Van Krevelen diagrams (H/C versus (O–3S)/C) for ambient OS formulas, colored by double bond equivalent (DBE). Because the sulfate group contributes three additional oxygen atoms compared to common oxygen-containing groups (e.g., carbonyl and hydroxyl groups), using (O–3S)/C ratio instead of the conventional O/C ratio more accurately reflects their degree of oxidation.^22^ Most OS formulas fall in the unsaturated-aliphatic domain across a wide span of (O–3S)/C. Notably, the distribution extends up to 1.5 because increasing nonsulfate oxygen content raises the (O–3S)/C ratio, indicating extensive oxidative processing of the organic moiety. Figure 1b and c separates OS formulas with suggested precursors from those that remain unclassified. Both groups occupy similar regions of Van Krevelen space, but the unclassified set exhibits a longer tail toward lower H/C values, implying a higher degree of unsaturation. Figure 1d highlights OS formulas that have been identified as OS products in laboratory transformation experiments, along with the subset of unclassified ambient OS formulas that can be explained by the transformation processes. The clustering of these points within the unsaturated-aliphatic band further supports the notion that many unclassified OSs could be originated from chemical transformation processes. Figure 1e shows the carbon-number distribution of classified and unclassified OSs. The largest number of ambient OS molecular formulas falls within the C_7_ to C_12_ carbon number range. Notably, unclassified OSs constitute a substantial fraction at higher carbon numbers and molecular weights, suggesting that many of these compounds (likely oligomers) have largely unexplored formation and transformation pathways. Taken together, the chemical space occupied by ambient OSs provides the hint that atmospheric transformation or chemical aging processes may represent a potential driver of their molecular diversity and degree of oxygenation.

While the OS formation pathways have been more extensively studied,^1,18,19,23–25^ their atmospheric transformation pathways beyond formation remain poorly understood and constrained (Scheme 1).^23,26^ It is not surprising that many freshly formed OSs are expected to continue evolving in the atmosphere. More recently, laboratory studies have demonstrated that certain atmospherically important OSs (e.g., 2-methyltetrol sulfates (2-MTS) and α-pinene-derived OSs) can undergo rapid and efficient transformations, including multiphase heterogeneous oxidation reactions near aerosol surface,^27–30^ aqueous-phase bulk oxidation reactions occurring within wet aerosols and cloud/fog droplets,^24,26,31–36^ and gas-phase oxidation of semivolatile OSs such as glycolic acid sulfate.^34^ Such chemical aging and conversion processes have been found to efficiently redistribute OS-bound sulfur and carbon, generating a large variety of higher-generation OS species, nonsulfated organic compounds, and inorganic sulfate (Scheme 1). Although these laboratory investigations of OS transformation are still very limited at present (only seven studies focused on product analysis, Table S1), they have identified 61 distinct OS formulas as transformation products, representing about 17% of the total ambient OS formulas (Figure 1b). Notably, among 236 unclassified ambient OS formulas, 19 (approximately 5%) can be directly matched to these laboratory-generated transformation products, demonstrating that transformation processes can potentially explain a fraction of previously unassigned OSs (Figure 1b and d). We also hypothesize that in addition to OSs freshly formed from photochemical reactions from different precursors, secondary or higher-generation OS species arising from the atmospheric transformation of these freshly formed OSs can potentially contribute to ambient OSs.

**Scheme 1:**
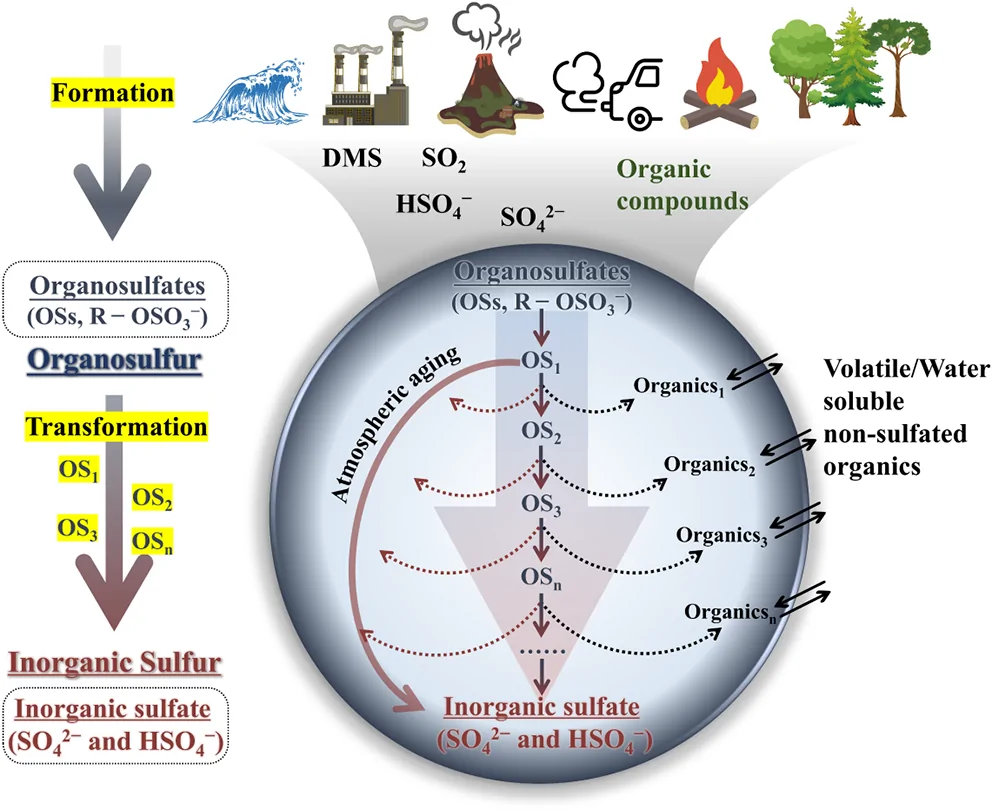
Proposed Generalized Pathways for Inorganic Sulfate Formation during the Atmospheric Aging of Organosulfates (OSs)^a^

a “OS_1_”and “Organics_1_”denote the first-generation OS and organic products formed from the transformation of OSs freshly formed from their precursors and inorganic sulfur species, with subsequent generations labeled accordingly.

We also note that several small and atmospherically abundant OSs, such as hydroxyacetone sulfate (C_3_H_5_SO_5_^**–**^), have commonly been attributed to isoprene oxidation in field studies (Table S1). For example, chamber experiments have reported its formation during photochemical oxidation and/or ozonolysis of isoprene in the presence of acidic sulfate seed aerosols.^18,37,38^ Sulfate-radical-induced oxidation in the bulk aqueous phase can also contribute to its formation from methacrolein and methyl vinyl ketone.^39^ More recently, Tiszenkel et al. also observed its presence during α-pinene ozonolysis in both the gas and particle phases.^10^ Furthermore, laboratory study has shown that aqueous-phase ^•^OH oxidation of α-pinene-derived OSs or propyl sulfate can yield smaller OSs, including hydroxyacetone sulfate, as a higher-generation product.^26,33^ These findings indicate that hydroxyacetone sulfate, and especially those small OSs could be originated from multiple VOC precursors via both formation and/or transformation pathways.

Currently, both field studies and atmospheric models largely neglect the transformation processes of OSs and focus mostly on precursor-specific formation pathways (Table S1). Even the formation of freshly generated OSs remains incompletely represented in atmospheric models for most precursor systems, with isoprene-derived OS formation via condensed-phase IEPOX chemistry in acidic sulfate aerosols being one of the few explicitly treated mechanisms.^40^ Consequently, estimates of OS concentrations and sources may be biased. For example, certain atmospheric OSs are often treated as relatively stable products and used as molecular tracers for specific VOC precursors. One example is 2-methylglyceric acid sulfate (C_4_H_7_SO_7_^**–**^), which has been widely used as a tracer for isoprene-derived secondary organic aerosol (SOA) formed through photochemical reactions under high-NO_*x*_ conditions.^41,42^ However, recent laboratory evidence indicates that this tracer can also be produced during atmospheric aging of 2-MTS via heterogeneous oxidation.^30^ Moreover, since transformation processes are not considered in most current field studies when qualifying OSs, the currently reported concentrations for certain OSs could be biased if their atmospheric transformation pathways are efficient and have not been corrected. Although incorporating the transformation processes of freshly generated OSs into atmospheric models remains challenge, it is essential for improving future predictions of OS abundance, sources, and the atmospheric sulfur cycle.

Compared with freshly formed OSs, the chemical stability, reaction kinetics, mechanisms, and fate of higher-generation OSs formed by transformation remain largely unconstrained. Laboratory evidence further shows that conversion of OS-bound sulfur to inorganic sulfate during chemical aging can be rapid.^26^ As shown in Scheme 1, these may also indicate that OSs are unlikely to represent the final products. Instead, they could undergo continuous transformation, ultimately mineralizing to inorganic sulfate. Detailed discussion of the evidence and mechanisms for conversion between organic and inorganic sulfur upon chemical aging of OSs is provided in Sections S2 and S3.

This perspective addresses that the current field investigations have mostly focused on OS formation pathways when qualifying and explaining OS sources and abundance, while the role of transformation pathways has received much less attention. This may limit our ability to fully account for the sources, diversity and abundance of OSs detected in the atmosphere. Atmospheric aging converts OS-bound sulfur into a diverse suite of new, higher-generation OSs, and ultimately into inorganic sulfate. These processes, together with OS formation pathways, likely governs the speciation and evolution of aerosol sulfur. This, in turn, influences aerosol physicochemical properties (e.g., surface tension, water uptake, acidity, and cloud activation activity),^43–45^ and their associated environmental and health impacts (e.g., climate forcing and oxidative stress and inflammation).^46,47^ An integrated framework that links laboratory studies, field observations, and atmospheric models would be much needed to investigate how chemical aging shapes the abundance, speciation, and fate of ambient OSs. Specifically, we recommend the following priorities: 1) investigating multigenerational chemical aging processes across diverse OSs using laboratory experiments and model simulations, quantifying their kinetics, reaction products, and mechanisms, particularly for higher-generation OS products formed through the successive transformation of freshly generated OSs under extended reaction time scales. As summarized in Table S1 and Figure 1e, a large number of unclassified ambient OSs have large carbon numbers and high molecular weights, suggesting they may be oligomers whose formation and transformation pathways remain largely unexplored. Future research should address their stability, reactivity, and whether they are primary or secondary in origins. This requires systematic measurement of rate constants for reactions of different OS classes (e.g., aliphatic, aromatic, nitrooxy-substituted) with key atmospheric oxidants (e.g., ^•^OH, SO_4_^•–^, NO_3_^•^, Cl^•^, and O_3_). Future studies should also move beyond single-component OS systems to investigate chemically complex multicomponent aerosols, investigating how aerosol composition, particle morphology, phase state, and the spatial distribution of OSs (e.g., surface-enrich versus bulk) influence OS transformation kinetics and reaction pathways. Complementary product studies should track the composition and yields of both organic and inorganic species via multiple aging progresses; 2) expanding laboratory chamber studies, authentic standard synthesis, and ultrahigh-resolution mass spectrometry coupled with suitable chromatographic techniques, including hydrophilic interaction liquid chromatography (HILIC) for resolving small, highly polar OSs (e.g., ≤C_5_ OSs) and reversed-phase liquid chromatography (RPLC) for larger, less polar OSs (e.g., monoterpene-derived OSs), together with MS/MS, is essential to achieve detailed molecular characterization, with particular emphasis on anthropogenic OSs.^48^ While current studies largely focus on biogenic OSs, anthropogenic sources such as aromatic hydrocarbons from combustion remain underexplored. Chamber experiments could simulate the aging of OSs under controlled conditions and yield insights transferable to the real atmosphere. Developing authentic standards and advancing ultrahigh-resolution mass spectrometry are important and urgent for accurate quantification and unambiguous identification in complex field samples, including oligomers and highly oxygenated species, thereby improving source apportionment and transformation analysis. Particular attention should also be given to resolving structural and stereoisomers of OSs, as isomer-specific identification is essential for accurately constraining their precursors, formation pathways, and atmospheric transformation processes. In parallel, there is an urgent need to develop quantitative real-time analytical methods for OS measurements. Integrating complementary offline techniques (e.g., HILIC/RPLC-MS/MS) with real-time aerosol mass spectrometric and chemical ionization approaches would enable more accurate characterization of OS formation and transformation while minimizing sampling artifacts associates with filter collection. Such developments are expected to improve our ability to quantify OS dynamics, resolve molecular structures, and investigate their transformation processes under atmospherically relevant conditions. 3) Establishing an aerosol sulfur mass balance for the interconversion between OS-bound sulfur and inorganic sulfate, particularly in laboratory studies, thereby better constraining the form and fate of aerosol sulfur upon aging. This further highlights the importance and urgency of addressing the quantification challenge, as quantitative measurements are essential for identifying the dominant OS products and transformation pathways and for closing the aerosol sulfur mass balance, and 4) broadening the focus of field observations to consider multiple sources, explicitly accounting for both formation and transformation pathways when quantifying OS concentrations and identifying their sources. Updated reaction schemes shall incorporate both new OS formation and transformation processes, constrained by model- and laboratory-derived parameters. Multiphase chemistry models should be continuously updated as new evidence on the particle-phase chemistry of OSs and its coupling to nonsulfur organics in both the particle phase but also the gas phase develops.

Overall, the atmospheric science community has made remarkable progress in understanding OS formation in the atmosphere. Increasing evidence has shown that ambient OSs are not stable end products but continue to undergo transforms after their initial formation. Because both OS formation and transformation remain important yet incompletely understood processes, elucidating these processes is essential for more accurately predicting the abundance, sources, composition, and environmental impacts of atmospheric OSs, particle sulfate, particle and gas phase organic compounds.

## Supplementary Material



## Notes

Summary of ambient field observations of OSs and laboratory studies of their transformation; conversion between organic and inorganic sulfur during the chemical aging of OSs; and detailed mechanisms for OS-bound sulfur conversion to inorganic sulfate (PDF)
