# Supplementary material for: What Are the Sources of Unclassified Organosulfates in the Atmosphere?
Source: Environ Sci Technol. 2026 Sep 1;60(36):25185–91. doi: 10.1021/acs.est.6c08394 (PMC13585059; doi:10.1021/acs.est.6c08394)
Supplement: Supplementary file 1 [file es6c08394_si_001.pdf]

Supplement of

What Are the Sources of Unclassified Organosulfates in the Atmosphere?

Donger Lai[1*], Thomas Schaefer[2], Ru-Jin Huang[3,4,5], Jian Zhen Yu[6,7], Hartmut Herrmann[2,8,9], and Man Nin Chan[1,10*]

[1]Department of Earth and Environmental Sciences, Faculty of Science, The Chinese University of Hong Kong, Hong Kong 999077, China

[2]Atmospheric Chemistry Department (ACD), Leibniz Institute for Tropospheric Research (TROPOS), Leipzig 04318, Germany

[3]State Key Laboratory of Loess Science, Institute of Earth Environment, Chinese Academy of Sciences, Xi'an 710061, China

[4]Institute of Global Environmental Change, Xi'an Jiaotong University, Xi'an 710049, China

[5]University of Chinese Academy of Sciences, Beijing 100049, China

[6]Division of Environment and Sustainability, The Hong Kong University of Science and Technology, Hong Kong 999077, China

[7]Department of Chemistry, The Hong Kong University of Science and Technology, Hong Kong 999077, China

[8]School of Environmental Science and Engineering, Shandong University, Qingdao 266237, China

[9]Shanghai Key Laboratory of Atmospheric Particle Pollution and Prevention, Department of Environmental Science & Engineering, Institute of Atmospheric Sciences, Fudan University, Shanghai 200433, China

[10]The Institute of Environment, Energy, and Sustainability, The Chinese University of Hong Kong, Hong Kong 999077, China

[*]To whom correspondence should be addressed:

Donger Lai: dongerlai@cuhk.edu.hk; Man Nin Chan: mnchan@cuhk.edu.hk

Section S1. Summary of ambient field observations of organosulfates (OSs) and laboratory studies on their transformation.

Table S1. Summary of ambient field observations of OSs and laboratory studies on their transformation.

OS compounds		Field observation							Laboratory studies on OS transformation	
m/z for $[M-H]^-$	Formula for $[M-H]^-$	Suggested precursors	Considers aging as source?	(O-3S)/C	Double bond equivalent (DBE)	Aromaticity equivalent (*Xc*)	OSc	Ref.	Suggested new formation pathway from transformation	Ref.
110.98	$CH_3SO_4^-$	Methanol*	no	1.00	0	0.00	5.00	1, 2	–	–
122.98	$C_2H_3SO_4^-$	–	no	0.50	1	1.00	2.50	3, 4	–	–
124.99	$C_2H_5SO_4^-$	–*	no	0.50	0	0.00	1.50	5	–	–
136.99	$C_3H_5SO_4^-$	Diesel	no	0.33	1	1.00	1.00	1, 6-9	–	–
138.97	$C_2H_3SO_5^-$	Isoprene, Glycolaldehyde, Hydroxyacetone	no	1.00	1	-1.00	3.50	2, 6-14	Aqueous-phase •OH oxidation of phenyl sulfate; Heterogeneous •OH oxidation of ethyl sulfate/ isoprene epoxydiol-derived methyltetrol sulfates	15-17
139.01	$C_3H_7SO_4^-$	–*	no	0.33	0	0.00	0.33	4	–	–
140.99	$C_2H_5SO_5^-$	Ethylene	no	1.00	0	7.00	2.50	2, 14, 18	Heterogeneous •OH oxidation of ethyl sulfate	16
151.01	$C_4H_7SO_4^-$	Diesel fuel	no	0.25	1	1.00	0.25	1, 3, 4, 6, 7, 9, 19, 20	–	–
152.99	$C_3H_5SO_5^-$	Isoprene, Methacrolein, Methyl vinyl ketone, Hydroxyacetone, Glycolaldehyde, α-Pinene, Anthropogenic	no	0.67	1	-1.00	1.67	1, 2, 4, 6-10, 12-14, 18, 19, 21-28	Aqueous-phase •OH oxidation of α-pinene-derived organosulfate/ propyl sulfate	29, 30
153.02	$C_4H_9SO_4^-$	–*	no	0.25	0	0.00	-0.25	4	–	–

154.97	$C_2H_3SO_6^-$	Glycolic acid, Isoprene, Methyl vinyl ketone, Hydroxyacetone, Glyoxal, Hydroxyacetic acid	no	1.50	1	0.00	4.50	2, 4, 6-11, 13, 14, 18, 19, 21-23, 26, 27, 31, 32	Heterogeneous $^{\bullet}$OH oxidation of isoprene epoxydiol-derived methyltetrol sulfates	17
155.00	$C_3H_7SO_5^-$	–	no	0.67	0	7.00	1.00	1, 2, 6-9	–	–
164.99	$C_4H_5SO_5^-$	–	no	0.50	2	1.67	1.25	4, 6, 7	–	–
165.02	$C_5H_9SO_4^-$	–	no	0.20	1	1.00	-0.20	3, 4	–	–
167.00	$C_4H_7SO_5^-$	Methacrolein, Methyl vinyl ketone	no	0.50	1	-1.00	0.75	2, 6, 7, 9, 10, 12, 13	–	–
167.04	$C_5H_{11}SO_4^-$	–*	no	0.20	0	0.00	-0.60	3, 4, 7, 33	–	–
168.98	$C_3H_5SO_6^-$	Lactic acid, Isoprene, 3-E-hexenal, 3-Z-hezenal, 2-E-pentenal, Methyl vinyl ketone, Methylglycolic acid, Methylglyoxal, 2-hydroxy propanoic acid, Hexenal, Anthropogenic, Green Leaf Volatiles	no	1.00	1	0.00	2.33	2, 4, 6-11, 13, 14, 18, 19, 21-23, 25, 27, 31, 32	Heterogeneous $^{\bullet}$OH oxidation of isoprene epoxydiol-derived methyltetrol sulfates	17
169.02	$C_4H_9SO_5^-$	–	no	0.50	0	7.00	0.25	4	–	–
171.00	$C_3H_7SO_6^-$	Isoprene	no	1.00	0	5.00	1.67	2, 4	Heterogeneous $^{\bullet}$OH oxidation of isoprene epoxydiol-derived methyltetrol sulfates; Aqueous-phase $^{\bullet}$OH oxidation of propyl sulfate	17, 30
172.99	$C_6H_5SO_4^-$	Naphthalene, 2-Methylnaphthalene	no	0.17	4	2.50	0.50	4, 7, 9, 20, 26, 34	–	–

179.00	$C_5H_7SO_5^-$	Isoprene	no	0.40	2	1.67	0.60	4, 12, 13	–	–
179.04	$C_6H_{11}SO_4^-$	–	no	0.17	1	1.00	-0.50	4	–	–
180.98	$C_4H_5SO_6^-$	Methacrolein	no	0.75	2	1.00	1.75	4, 13	–	–
181.02	$C_5H_9SO_5^-$	2-methylglyceric acid, Isoprene	no	0.40	1	-1.00	0.20	4, 10, 12, 22, 35	–	–
181.05	$C_6H_{13}SO_4^-$	–*	no	0.17	0	0.00	-0.83	4, 7	–	–
183.00	$C_4H_7SO_6^-$	Isoprene, Methacrolein, Methyl vinyl ketone, Anthropogenic	no	0.75	1	0.00	1.25	1, 2, 4, 6, 7, 9, 10, 13, 14, 18, 23, 25, 36	–	–
183.03	$C_5H_{11}SO_5^-$	–	no	0.40	0	7.00	-0.20	4	–	–
184.98	$C_3H_5SO_7^-$	Isoprene	no	1.33	1	7.00	3.00	2, 4	Heterogeneous •OH oxidation of isoprene epoxydiol-derived methyltetrol sulfates	17
184.99	$C_7H_5SO_4^-$	Aromatics (Benzene and PAHs)	no	0.14	5	2.60	0.43	31	–	–
187.01	$C_7H_7SO_4^-$	Benzene, Aromatics, 2-Methylnaphthalene	no	0.14	4	2.50	0.14	4, 7, 9, 14, 20, 24, 26, 31, 34	–	–
188.99	$C_6H_5SO_5^-$	–	no	0.33	4	2.43	0.83	4	Aqueous-phase •OH oxidation of phenyl sulfate	15, 30
191.04	$C_7H_{11}SO_4^-$	–	no	0.14	2	2.00	-0.43	4	–	–
192.98	$C_5H_5SO_6^-$	–	no	0.60	3	2.00	1.40	4	Aqueous-phase •OH oxidation of phenyl sulfate	15
193.02	$C_6H_9SO_5^-$	–	no	0.33	2	1.67	0.17	4	–	–
193.05	$C_7H_{13}SO_4^-$	–	no	0.14	1	1.00	-0.71	4	–	–
195.00	$C_5H_7SO_6^-$	Diesel and biodiesel fuel, Isoprene	no	0.60	2	1.00	1.00	1, 4, 6, 7, 9, 13, 20, 25, 35	–	–

195.03	$C_6H_{11}SO_5^-$	–	no	0.33	1	-1.00	-0.17	4, 12, 35	–	–
195.07	$C_7H_{15}SO_4^-$	Anthropogenic*	no	0.14	0	0.00	-1.00	4, 7, 20, 33	–	–
197.01	$C_5H_9SO_6^-$	Isoprene	no	0.60	1	0.00	0.60	2, 4, 6, 7, 9, 13, 14, 18, 25, 35	–	–
197.05	$C_6H_{13}SO_5^-$	–	no	0.33	0	7.00	-0.50	4	–	–
198.99	$C_4H_7SO_7^-$	Isoprene, Methyl vinyl ketone, Methacrolein, 2-methylglyceric acid, 2-methyl-3-buten-2-ol, Methacrylic Acid Epoxide	no	1.00	1	7.00	1.75	2, 4, 6, 7, 9-11, 13, 18, 19, 21-23, 25, 27, 36-38	Heterogeneous •OH oxidation of isoprene epoxydiol-derived methyltetrol sulfates	17
199.01	$C_8H_7SO_4^-$	Aromatics (Benzene and PAHs)	no	0.13	5	2.60	0.13	20, 31	–	–
199.03	$C_5H_{11}SO_6^-$	Isoprene, 2-methyl-3-buten-2-ol	no	0.60	0	5.00	0.20	1, 2, 6, 7, 9, 13, 21, 22	–	–
200.00	$C_4H_8SO_7^-$	2-methylglyceric acid (isoprene)	no	1.00	1	5.00	1.50	31	–	–
200.99	$C_7H_5SO_5^-$	2-Methylnaphthalene	no	0.29	5	2.56	0.71	4, 20, 35	–	–
201.02	$C_8H_9SO_4^-$	Benzene	no	0.13	4	2.50	-0.13	4, 31, 34	–	–
205.02	$C_7H_9SO_5^-$	–	no	0.29	3	2.20	0.14	4	–	–
205.05	$C_8H_{13}SO_4^-$	–	no	0.13	2	2.00	-0.63	4	–	–
207.00	$C_6H_7SO_6^-$	–	no	0.50	3	2.00	0.83	4, 12, 22	–	–
207.03	$C_7H_{11}SO_5^-$	–	no	0.29	2	1.67	-0.14	4, 25	–	–
207.07	$C_8H_{15}SO_4^-$	–	no	0.13	1	1.00	-0.88	4, 25, 35	–	–
209.01	$C_6H_9SO_6^-$	Diesel, Biodiesel fuel, Cyclohexene	no	0.50	2	1.00	0.50	4, 6, 7, 9, 18, 20, 33, 35	Aqueous-phase •OH oxidation of α-pinene-derived organosulfate	29

209.05	$C_7H_{13}SO_5^-$	Dodecane, Alkane, Anthropogenic	no	0.29	1	-1.00	-0.43	3, 4, 10, 12, 19, 20, 22, 25, 33, 35, 39	Heterogeneous $^{\bullet}$OH oxidation of dodecyl sulfate	40
209.08	$C_8H_{17}SO_4^-$	Anthropogenic*	no	0.13	0	0.00	-1.13	4, 7, 9, 20, 22, 33	–	–
210.99	$C_5H_7SO_7^-$	Isoprene, 3-Z-hexenal	no	0.80	2	-1.00	1.40	1, 2, 4, 6, 7, 9-11, 13, 14, 18, 19, 21, 22, 27, 36, 37, 41	Heterogeneous $^{\bullet}$OH oxidation of isoprene epoxydiol-derived methyltetrol sulfates; Aqueous-phase $^{\bullet}$OH oxidation of α-pinene-derived organosulfate	17, 29
211.03	$C_6H_{11}SO_6^-$	–	no	0.50	1	0.00	0.17	4, 25, 33, 35	Heterogeneous $^{\bullet}$OH oxidation of dodecyl sulfate	40
211.06	$C_7H_{15}SO_5^-$	Anthropogenic	no	0.29	0	7.00	-0.71	4, 20, 25	–	–
212.00	$C_5H_8SO_7^-$	Isoprene	no	0.80	2	0.00	1.20	31	–	–
212.99	$C_8H_5SO_5^-$	–	no	0.25	6	2.64	0.63	7, 22	–	–
213.01	$C_5H_9SO_7^-$	Isoprene, Fatty acid	no	0.80	1	7.00	1.00	1, 2, 4, 6, 7, 9, 10, 13, 18, 21-25, 36, 37, 42	Heterogeneous $^{\bullet}$OH oxidation of isoprene epoxydiol-derived methyltetrol sulfates	17
213.02	$C_9H_9SO_4^-$	–	no	0.11	5	2.60	-0.11	4, 20	–	–
215.00	$C_8H_7SO_5^-$	2-Methylnaphthalene	no	0.25	5	2.56	0.38	4, 20, 35	–	–
215.02	$C_5H_{11}SO_7^-$	Isoprene, IEPOX, 2-methyltetrols, α-Pinene, 3-Z-hexenal	no	0.80	0	4.33	0.60	1-4, 6, 7, 9-11, 13, 18, 19, 21-25, 27,	–	–

								31, 36-38, 41-43		
216.98	$C_7H_5SO_6^-$	–	no	0.43	5	2.50	1.00	4	–	–
217.02	$C_8H_9SO_5^-$	–	no	0.25	4	2.43	0.13	4	–	–
219.03	$C_8H_{11}SO_5^-$	–	no	0.25	3	2.20	-0.13	4	–	–
219.07	$C_9H_{15}SO_4^-$	–	no	0.11	2	2.00	-0.78	4	–	–
221.01	$C_7H_9SO_6^-$	–	no	0.43	3	2.00	0.43	4	–	–
221.05	$C_8H_{13}SO_5^-$	–	no	0.25	2	1.67	-0.38	3, 4	–	–
221.08	$C_9H_{17}SO_4^-$	Anthropogenic	no	0.11	1	1.00	-1.00	4, 20, 22, 35	–	–
222.99	$C_6H_7SO_7^-$	–	no	0.67	3	1.67	1.17	4	Aqueous-phase $^{\bullet}OH$ oxidation of phenyl sulfate; α-pinene-derived organosulfate	15, 29
223.03	$C_7H_{11}SO_6^-$	Monoterpenes, α-Pinene	no	0.43	2	1.00	0.14	1, 2, 4, 6, 7, 9, 18, 24, 25, 35	–	–
223.06	$C_8H_{15}SO_5^-$	Anthropogenic, Diesel fuel	no	0.25	1	-1.00	-0.63	3, 4, 12, 19, 20, 35	Heterogeneous $^{\bullet}OH$ oxidation of dodecyl sulfate	40
223.10	$C_9H_{19}SO_4^-$	Anthropogenic*	no	0.11	0	0.00	-1.22	4, 7, 20, 22	–	–
224.97	$C_5H_5SO_8^-$	Isoprene	no	1.00	3	1.00	2.20	13	–	–
225.01	$C_6H_9SO_7^-$	Isoprene, Cyclohexene, Hexenal, 2-Methylnaphthalene, Green leaf volatiles	no	0.67	2	-1.00	0.83	2, 4, 18, 25, 35, 41	–	–
225.04	$C_7H_{13}SO_6^-$	–	no	0.43	1	0.00	-0.14	3, 4, 19, 20, 33, 35	Heterogeneous $^{\bullet}OH$ oxidation of dodecyl sulfate	40
225.08	$C_8H_{17}SO_5^-$	–	no	0.25	0	7.00	-0.88	4	–	–
226.01	$C_6H_{10}SO_7^-$	–	no	0.67	2	0.00	0.67	31	–	–

226.99	$C_5H_7SO_8^-$	Isoprene	no	1.00	2	0.00	1.80	2, 14, 41	Heterogeneous •OH oxidation of isoprene epoxydiol-derived methyltetrol sulfates; Aqueous-phase •OH oxidation of phenyl sulfate	15, 17
227.00	$C_9H_7SO_5^-$	2-Methylnaphthalene	no	0.22	6	2.64	0.33	4	–	–
227.02	$C_6H_{11}SO_7^-$	Isoprene	no	0.67	1	7.00	0.50	4	–	–
227.04	$C_{10}H_{11}SO_4^-$	–	no	0.10	5	2.60	-0.30	4	–	–
227.06	$C_7H_{15}SO_6^-$	–	no	0.43	0	5.00	-0.43	4	–	–
228.98	$C_8H_5SO_6^-$	–	no	0.38	6	2.60	0.88	20	–	–
229.00	$C_5H_9SO_8^-$	Isoprene, Green leaf volatiles	no	1.00	1	5.00	1.40	2, 4, 13	Heterogeneous •OH oxidation of isoprene epoxydiol-derived methyltetrol sulfates	17
229.02	$C_9H_9SO_5^-$	Aromatics (Benzene and PAHs), 2-Methylnaphthalene	no	0.22	5	2.56	0.11	4, 31	–	–
231.00	$C_8H_7SO_6^-$	–	no	0.38	5	2.50	0.63	4	–	–
231.02	$C_5H_{11}SO_8^-$	Isoprene	no	1.00	0	4.00	1.00	2	Heterogeneous •OH oxidation of isoprene epoxydiol-derived methyltetrol sulfates	17
231.03	$C_9H_{11}SO_5^-$	Aromatics (Benzene and PAHs), 2-methylnaphthalene	no	0.22	4	2.43	-0.11	22, 31, 44	–	–
231.07	$C_{10}H_{15}SO_4^-$	α-Pinene	no	0.10	3	2.33	-0.70	28, 45	–	–
233.01	$C_8H_9SO_6^-$	–	no	0.38	4	2.33	0.38	4	Aqueous-phase •OH oxidation of α-pinene-derived organosulfate	29
233.05	$C_9H_{13}SO_5^-$	–	no	0.22	3	2.20	-0.33	4	–	–
234.06	$C_9H_{14}SO_5^-$	–	no	0.22	3	2.00	-0.44	42	–	–
235.03	$C_8H_{11}SO_6^-$	–	no	0.38	3	2.00	0.13	4	–	–

235.06	$C_9H_{15}SO_5^-$	Anthropogenic, Diesel fuel	no	0.22	2	1.67	-0.56	3, 4, 19, 20, 25, 33, 35	Aqueous-phase •OH oxidation of α-pinene-derived organosulfate	29
235.10	$C_{10}H_{19}SO_4^-$	–	no	0.10	1	1.00	-1.10	4	–	–
237.01	$C_7H_9SO_7^-$	Isoprene, Methyl vinyl ketone	no	0.57	3	1.67	0.71	1, 2, 4, 6, 7, 9, 25, 35	Aqueous-phase •OH oxidation of α-pinene-derived organosulfate	29
237.04	$C_8H_{13}SO_6^-$	Monoterpenes, α-Pinene, Anthropogenic, Diesel fuel	no	0.38	2	1.00	-0.13	3, 4, 18-20, 24, 33, 35	–	–
237.08	$C_9H_{17}SO_5^-$	Anthropogenic, Alkane, Dodecane	no	0.22	1	-1.00	-0.78	3, 4, 10, 19, 20, 22, 25, 39	Heterogeneous •OH oxidation of dodecyl sulfate	40
237.12	$C_{10}H_{21}SO_4^-$	Anthropogenic*	no	0.10	0	0.00	-1.30	3, 4, 7, 20, 22	–	–
239.02	$C_7H_{11}SO_7^-$	Monoterpenes, Limonene, Methyl vinyl ketone, Methacrolein,	no	0.57	2	-1.00	0.43	1, 2, 4, 6, 7, 9, 14, 18, 25, 33, 35, 43	–	–
239.06	$C_8H_{15}SO_6^-$	Anthropogenic, Diesel fuel	no	0.38	1	0.00	-0.38	3, 4, 19, 35	Heterogeneous •OH oxidation of dodecyl sulfate	40
239.10	$C_9H_{19}SO_5^-$	–	no	0.22	0	7.00	-1.00	3	–	–
240.97	$C_5H_5SO_9^-$	Isoprene	no	1.20	3	-1.00	2.60	13	–	–
241.00	$C_6H_9SO_8^-$	–	no	0.83	2	0.00	1.17	4, 39	Aqueous-phase •OH oxidation of α-pinene-derived organosulfate	29
241.02	$C_{10}H_9SO_5^-$	–	no	0.20	6	2.64	0.10	4	–	–
241.04	$C_7H_{13}SO_7^-$	–	no	0.57	1	7.00	0.14	4	–	–
241.05	$C_{11}H_{13}SO_4^-$	–	no	0.09	5	2.60	-0.45	4	–	–

241.07	$C_8H_{17}SO_6^-$	–	no	0.38	0	5.00	-0.63	4	–	–
242.98	$C_5H_7SO_9^-$	Isoprene	no	1.20	2	7.00	2.20	13	–	–
243.00	$C_9H_7SO_6^-$	–	no	0.33	6	2.60	0.56	4, 20	–	–
243.03	$C_{10}H_{11}SO_5^-$	–	no	0.20	5	2.56	-0.10	4	–	–
245.00	$C_5H_9SO_9^-$	α-Pinene	no	1.20	1	4.33	1.80	**28**	–	–
245.01	$C_9H_9SO_6^-$	–	no	0.33	5	2.50	0.33	4, 20	–	–
245.05	$C_{10}H_{13}SO_5^-$	–	no	0.20	4	2.43	-0.30	4	–	–
247.03	$C_9H_{11}SO_6^-$	–	no	0.33	4	2.33	0.11	4	–	–
247.06	$C_{10}H_{15}SO_5^-$	Monoterpenes, α-Pinene	no	0.20	3	2.20	-0.50	3, 4, 10, 22, 24, 35	Aqueous-phase •OH oxidation of α-pinene-derived Organosulfate; Heterogeneous •OH oxidation of α-pinene-derived Organosulfate	29, 46
249.04	$C_9H_{13}SO_6^-$	Monoterpenes, Terpinolene, Limonene, Isoprene	no	0.33	3	2.00	-0.11	3, 4, 35	Aqueous-phase •OH oxidation of α-pinene-derived organosulfate	29
249.08	$C_{10}H_{17}SO_5^-$	Monoterpenes, α, β-Pinene, Limonene, Terpinolene, Anthropogenic	no	0.20	2	1.67	-0.70	3, 4, 7, 9, 10, 12, 14, 19-22, 24, 27, 28, 33, 35, 42, 45, 47	–	–
249.12	$C_{11}H_{21}SO_4^-$	–	no	0.09	1	1.00	-1.18	4	–	–
251.02	$C_8H_{11}SO_7^-$	Methyl vinyl ketone	no	0.50	3	1.67	0.38	2, 4, 35, 41	Aqueous-phase •OH oxidation of α-pinene-derived organosulfate	29

251.06	$C_9H_{15}SO_6^-$	Monoterpene, α-Pinene, Limonene, β-caryophyllene, Limonaketone, Sesquiterpene, Anthropogenic	no	0.33	2	1.00	-0.33	3, 4, 6, 7, 9, 10, 18-22, 25, 27, 28, 33, 35, 47	Aqueous-phase $^{•}$OH oxidation of α-pinene-derived organosulfate	29
251.10	$C_{10}H_{19}SO_5^-$	Monoterpene, Anthropogenic, Cyclodecane, Alkane	no	0.20	1	-1.00	-0.90	3, 4, 7, 19, 20, 22, 35, 39, 45	–	–
251.13	$C_{11}H_{23}SO_4^-$	–*	no	0.09	0	0.00	-1.36	3, 4, 7	–	–
253.04	$C_8H_{13}SO_7^-$	Monoterpenes, α-Terpinene, Methyl vinyl ketone, Methacrolein	no	0.50	2	-1.00	0.13	4, 6, 7, 9, 14, 18, 22, 33, 35, 48	–	–
253.07	$C_9H_{17}SO_6^-$	–	no	0.33	1	0.00	-0.56	3, 4, 19, 20, 33, 35	Heterogeneous $^{•}$OH oxidation of dodecyl sulfate	40
253.11	$C_{10}H_{21}SO_5^-$	–	no	0.20	0	7.00	-1.10	3	–	–
255.02	$C_7H_{11}SO_8^-$	Isoprene	no	0.71	2	0.00	0.71	4, 35	Aqueous-phase $^{•}$OH oxidation of α-pinene-derived organosulfate	29
255.03	$C_{11}H_{11}SO_5^-$	–	no	0.18	6	2.64	-0.09	4	–	–
255.05	$C_8H_{15}SO_7^-$	–	no	0.50	1	7.00	-0.13	4	–	–
255.09	$C_9H_{19}SO_6^-$	–	no	0.33	0	5.00	-0.78	4	–	–
257.01	$C_{10}H_9SO_6^-$	Naphthalene	no	0.30	6	2.60	0.30	4, 7, 44	–	–
257.05	$C_{11}H_{13}SO_5^-$	–	no	0.18	5	2.56	-0.27	4	–	–
258.99	$C_9H_7SO_7^-$	–	no	0.44	6	2.56	0.78	4, 20	–	–
259.03	$C_{10}H_{11}SO_6^-$	–	no	0.30	5	2.50	0.10	4	–	–
261.04	$C_{10}H_{13}SO_6^-$	–	no	0.30	4	2.33	-0.10	4	–	–
261.08	$C_{11}H_{17}SO_5^-$	–	no	0.18	3	2.20	-0.64	4	–	–

263.02	$C_9H_{11}SO_7^-$	Monoterpenes	no	0.44	4	2.20	0.33	4, 22	Aqueous-phase •OH oxidation of α-pinene-derived organosulfate	29
263.06	$C_{10}H_{15}SO_6^-$	Monoterpenes, α, β-Pinene	no	0.30	3	2.00	-0.30	3, 4, 22, 24, 28, 35	Aqueous-phase •OH oxidation of α-pinene-derived Organosulfate; Heterogeneous •OH oxidation of α-pinene-derived Organosulfate	29, 46
263.10	$C_{11}H_{19}SO_5^-$	–	no	0.18	2	1.67	-0.82	3, 4, 33	–	–
265.00	$C_8H_9SO_8^-$	–	no	0.63	4	2.00	0.88	22	–	–
265.02	$C_{12}H_9SO_5^-$	–	no	0.17	8	2.73	0.08	4	–	–
265.04	$C_9H_{13}SO_7^-$	–	no	0.44	3	1.67	0.11	4, 7, 22, 33, 35, 45	Aqueous-phase •OH oxidation of α-pinene-derived organosulfate	29
265.07	$C_{10}H_{17}SO_6^-$	Monoterpenes, Terpinolene, α-Terpinene, α-Pinene, Anthropogenic, Alkane, Cyclodecane	no	0.30	2	1.00	-0.50	3, 4, 19, 20, 22, 24, 28, 35, 39, 45	Aqueous-phase •OH oxidation of α-pinene-derived Organosulfate; Heterogeneous •OH oxidation of α-pinene-derived Organosulfate	29, 46
265.11	$C_{11}H_{21}SO_5^-$	Anthropogenic	no	0.18	1	-1.00	-1.00	3, 4, 7, 20, 22, 35	–	–
265.15	$C_{12}H_{25}SO_4^-$	Anthropogenic, Diesel, Biodiesel fuel*	no	0.08	0	0.00	-1.42	1, 2, 6, 18, 22	–	–
267.02	$C_8H_{11}SO_8^-$	Isoprene	no	0.63	3	1.00	0.63	4, 22, 25, 33, 35	Aqueous-phase •OH oxidation of α-pinene-derived	29

									organosulfate	
267.05	$C_9H_{15}SO_7^-$	Monoterpenes, Limonene, α-Pinene, Alkane, Isoprene, Anthropogenic, Decalin	no	0.44	2	-1.00	-0.11	3, 4, 10, 14, 18-20, 22, 25, 33, 35, 39, 45, 48	Aqueous-phase •OH oxidation of α-pinene-derived organosulfate	29
267.09	$C_{10}H_{19}SO_6^-$	Monoterpenes, Anthropogenic, Diesel fuel	no	0.30	1	0.00	-0.70	3, 4, 19, 20, 22, 33, 35	Heterogeneous •OH oxidation of dodecyl sulfate	40
269.03	$C_8H_{13}SO_8^-$	Isoprene	no	0.63	2	0.00	0.38	4, 22, 33, 35	–	–
269.05	$C_{12}H_{13}SO_5^-$	–	no	0.17	6	2.64	-0.25	4	–	–
269.07	$C_9H_{17}SO_7^-$	Anthropogenic, Alkane, Decalin, Monoterpenes	no	0.44	1	7.00	-0.33	4, 10, 19, 20, 22, 33, 39	–	–
269.11	$C_{10}H_{21}SO_6^-$	–	no	0.30	0	5.00	-0.90	4, 22	–	–
271.01	$C_7H_{11}SO_9^-$	–	no	0.86	2	7.00	1.00	20	Aqueous-phase •OH oxidation of α-pinene-derived organosulfate	29
271.03	$C_{11}H_{11}SO_6^-$	–	no	0.27	6	2.60	0.09	4, 22	–	–
271.05	$C_8H_{15}SO_8^-$	–	no	0.63	1	5.00	0.13	22	–	–
271.06	$C_{12}H_{15}SO_5^-$	–	no	0.17	5	2.56	-0.42	4	–	–
272.99	$C_6H_9SO_{10}^-$	α-Pinene	no	1.17	2	5.00	1.83	**28**	–	–
273.01	$C_{10}H_9SO_7^-$	Naphthalene	no	0.40	6	2.56	0.50	4, 22	–	–
273.02	$C_{14}H_9SO_4^-$	–	no	0.07	10	2.80	-0.07	4	–	–
273.03	$C_7H_{13}SO_9^-$	Anthropogenic	no	0.86	1	4.33	0.71	20	–	–
273.04	$C_{11}H_{13}SO_6^-$	–	no	0.27	5	2.50	-0.09	4	–	–
275.02	$C_{10}H_{11}SO_7^-$	–	no	0.40	5	2.43	0.30	22	–	–
277.04	$C_{10}H_{13}SO_7^-$	–	no	0.40	4	2.20	0.10	4	Aqueous-phase •OH oxidation of α-pinene-derived	29, 46

									Organosulfate; Heterogeneous $^{\bullet}$OH oxidation of α-pinene-derived Organosulfate	
277.07	$C_{11}H_{17}SO_6^-$	–	no	0.27	3	2.00	-0.45	4	–	–
277.11	$C_{12}H_{21}SO_5^-$	–	no	0.17	2	1.67	-0.92	4	–	–
279.02	$C_9H_{11}SO_8^-$	–	no	0.56	4	2.00	0.56	4	–	–
279.05	$C_{10}H_{15}SO_7^-$	Monoterpenes, α, γ -Terpinene, α, β-Pinene, Limonene, Pinene, Anthropogenic, Alkane, Dodecane, Cyclododecane	no	0.40	3	1.67	-0.10	1-4, 6, 7, 9, 10, 12, 14, 18, 19, 21, 22, 24, 25, 28, 33, 35, 39, 42, 43, 45, 48	Aqueous-phase $^{\bullet}$OH oxidation of α-pinene-derived Organosulfate; Heterogeneous $^{\bullet}$OH oxidation of α-pinene-derived Organosulfate	29, 46
279.09	$C_{11}H_{19}SO_6^-$	Anthropogenic, Diesel fuel	no	0.27	2	1.00	-0.64	3, 4, 19, 20, 35	–	–
279.13	$C_{12}H_{23}SO_5^-$	Anthropogenic, Alkane, Dodecane	no	0.17	1	-1.00	-1.08	3, 4, 7, 10, 19, 20, 25, 35, 39	Heterogeneous $^{\bullet}$OH oxidation of dodecyl sulfate	40
279.16	$C_{13}H_{27}SO_4^-$	–*	no	0.08	0	0.00	-1.46	4, 22	–	–
281.00	$C_8H_9SO_9^-$	–	no	0.75	4	1.67	1.13	22	–	–
281.03	$C_9H_{13}SO_8^-$	Isoprene, Monoterpenes, α-Pinene	no	0.56	3	1.00	0.33	4, 22, 28, 33, 35, 45	Aqueous-phase $^{\bullet}$OH oxidation of α-pinene-derived organosulfate	29
281.07	$C_{10}H_{17}SO_7^-$	Monoterpenes, pinene, α, β-Pinene, α Terpinene, Limonene, T erpinolene, Diesel fuel, Alkane	no	0.40	2	-1.00	-0.30	1-4, 6, 7, 9, 10, 18, 19, 22, 24,	Aqueous-phase $^{\bullet}$OH oxidation of α-pinene-derived Organosulfate; Heterogeneous $^{\bullet}$OH	29, 46

								28, 33, 35, 43	oxidation of α-pinene-derived Organosulfate	
281.11	$C_{11}H_{21}SO_6^-$	Diesel fuel	no	0.27	1	0.00	-0.82	3, 4, 19, 22	–	–
281.14	$C_{12}H_{25}SO_5^-$	–	no	0.17	0	7.00	-1.25	22	Heterogeneous $^{\bullet}$OH oxidation of dodecyl sulfate	40
282.06	$C_{13}H_{14}SO_5^-$	–	no	0.15	7	2.67	-0.31	25	–	–
283.01	$C_8H_{11}SO_9^-$	–	no	0.75	3	-1.00	0.88	4, 22	Aqueous-phase $^{\bullet}$OH oxidation of α-pinene-derived organosulfate	29
283.05	$C_9H_{15}SO_8^-$	Monoterpenes, α-Terpinene, Diesel fuel	no	0.56	2	0.00	0.11	4, 19, 22, 25, 35	Aqueous-phase $^{\bullet}$OH oxidation of α-pinene-derived organosulfate	29
283.06	$C_{13}H_{15}SO_5^-$	–	no	0.15	6	2.64	-0.38	4	–	–
283.09	$C_{10}H_{19}SO_7^-$	Monoterpenes, α-Terpinene, Terpinolene, β-Pinene, Diesel fuel	no	0.40	1	7.00	-0.50	4, 19, 22, 24, 45	–	–
283.12	$C_{11}H_{23}SO_6^-$	–	no	0.27	0	5.00	-1.00	4, 22	–	–
285.03	$C_8H_{13}SO_9^-$	–	no	0.75	2	7.00	0.63	20, 22	–	–
285.04	$C_{12}H_{13}SO_6^-$	–	no	0.25	6	2.60	-0.08	4	–	–
285.06	$C_9H_{17}SO_8^-$	Decalin	no	0.56	1	5.00	-0.11	4, 22, 39	–	–
285.08	$C_{13}H_{17}SO_5^-$	–	no	0.15	5	2.56	-0.54	4	–	–
286.99	$C_{10}H_7SO_8^-$	–	no	0.50	7	2.60	0.90	22	–	–
287.01	$C_7H_{11}SO_{10}^-$	–	no	1.00	2	5.00	1.29	20	Aqueous-phase $^{\bullet}$OH oxidation of α-pinene-derived organosulfate	29
287.02	$C_{11}H_{11}SO_7^-$	Aromatics (Benzene and PAHs), 2-Methylnaphthalene	no	0.36	6	2.56	0.27	4, 22, 31	–	–

289.00	$C_{10}H_9SO_8^-$	–	no	0.50	6	2.50	0.70	22	–	–
289.04	$C_{11}H_{13}SO_7^-$	2-methylnaphthalene	no	0.36	5	2.43	0.09	22, 44	–	–
291.02	$C_{10}H_{11}SO_8^-$	–	no	0.50	5	2.33	0.50	22	–	–
291.03	$C_{14}H_{11}SO_5^-$	–	no	0.14	9	2.76	-0.07	22	–	–
291.05	$C_{11}H_{15}SO_7^-$	–	no	0.36	4	2.20	-0.09	4	–	–
291.09	$C_{12}H_{19}SO_6^-$	–	no	0.25	3	2.00	-0.58	3, 4	–	–
291.13	$C_{13}H_{23}SO_5^-$	–	no	0.15	2	1.67	-1.00	4	–	–
293.03	$C_{10}H_{13}SO_8^-$	–	no	0.50	4	2.00	0.30	4, 22, 35	Aqueous-phase •OH oxidation of α-pinene-derived organosulfate	29
293.07	$C_{11}H_{17}SO_7^-$	Diesel fuel	no	0.36	3	1.67	-0.27	3, 4, 19, 35	–	–
293.11	$C_{12}H_{21}SO_6^-$	Anthropogenic	no	0.25	2	1.00	-0.75	3, 4, 19, 20, 35	Heterogeneous •OH oxidation of dodecyl sulfate	40
293.14	$C_{13}H_{25}SO_5^-$	Anthropogenic	no	0.15	1	-1.00	-1.15	3, 4, 7, 20, 22, 35	–	–
293.18	$C_{14}H_{29}SO_4^-$	–*	no	0.07	0	0.00	-1.50	22, 25, 35, 45	–	–
295.05	$C_{10}H_{15}SO_8^-$	Monoterpenes, α-Pinene, Alkane, Decalin	no	0.50	3	1.00	0.10	4, 10, 19, 22, 28, 35, 39, 45	Aqueous-phase •OH oxidation of α-pinene-derived organosulfate	29
295.09	$C_{11}H_{19}SO_7^-$	Diesel fuel	no	0.36	2	-1.00	-0.45	3, 4, 19, 22, 33, 35	–	–
295.12	$C_{12}H_{23}SO_6^-$	Diesel fuel	no	0.25	1	0.00	-0.92	3, 4, 19, 22	Heterogeneous •OH oxidation of dodecyl sulfate	40
295.16	$C_{13}H_{27}SO_5^-$	–	no	0.15	0	7.00	-1.31	22	–	–
297.03	$C_9H_{13}SO_9^-$	Isoprene, Monoterpenes	no	0.67	3	-1.00	0.56	4, 35, 45	–	–

297.06	$C_{10}H_{17}SO_8^-$	Monoterpenes, α-Pinene, α-Terpinene, Terpinolene, Anthropogenic, Alkane, Decalin	no	0.50	2	0.00	-0.10	1, 4, 10, 19, 22, 25, 35, 39, 45, 48	Aqueous-phase •OH oxidation of α-pinene-derived organosulfate	29
297.10	$C_{11}H_{21}SO_7^-$	–	no	0.36	1	7.00	-0.64	4	–	–
299.04	$C_9H_{15}SO_9^-$	Monoterpenes	no	0.67	2	7.00	0.33	4, 22	–	–
299.08	$C_{10}H_{19}SO_8^-$	–	no	0.50	1	5.00	-0.30	4	–	–
299.13	$C_{15}H_{23}SO_4^-$	–	no	0.07	4	2.50	-1.00	4	–	–
301.02	$C_8H_{13}SO_{10}^-$	–	no	0.88	2	5.00	0.88	20, 22	–	–
301.04	$C_{12}H_{13}SO_7^-$	–	no	0.33	6	2.56	0.08	4	–	–
301.11	$C_{14}H_{21}SO_5^-$	–	no	0.14	4	2.43	-0.79	4	–	–
305.07	$C_{12}H_{17}SO_7^-$	–	no	0.33	4	2.20	-0.25	4	–	–
305.11	$C_{13}H_{21}SO_6^-$	–	no	0.23	3	2.00	-0.69	4	–	–
305.14	$C_{14}H_{25}SO_5^-$	–	no	0.14	2	1.67	-1.07	4	–	–
307.05	$C_{11}H_{15}SO_8^-$	–	no	0.45	4	2.00	0.09	4	–	–
307.09	$C_{12}H_{19}SO_7^-$	–	no	0.33	3	1.67	-0.42	3, 4, 19, 22, 35	Heterogeneous •OH oxidation of dodecyl sulfate	40
307.12	$C_{13}H_{23}SO_6^-$	–	no	0.23	2	1.00	-0.85	3, 4, 19, 35	–	–
307.16	$C_{14}H_{27}SO_5^-$	Anthropogenic, Diesel fuel	no	0.14	1	-1.00	-1.21	3, 4, 7, 19, 20, 22, 35	–	–
307.19	$C_{15}H_{31}SO_4^-$	–*	no	0.07	0	0.00	-1.53	4, 22, 35	–	–
309.03	$C_{10}H_{13}SO_9^-$	Monoterpenes	no	0.60	4	1.67	0.50	4, 22, 45	Aqueous-phase •OH oxidation of α-pinene-derived organosulfate	29
309.06	$C_{11}H_{17}SO_8^-$	–	no	0.45	3	1.00	-0.09	4, 35	–	–
309.10	$C_{12}H_{21}SO_7^-$	Anthropogenic, Diesel fuel	no	0.33	2	-1.00	-0.58	3, 4, 19, 20, 35	Heterogeneous •OH oxidation of dodecyl sulfate	40

309.14	$C_{13}H_{25}SO_6^-$	Anthropogenic	no	0.23	1	0.00	-1.00	3, 4, 20, 25, 35	–	–
309.17	$C_{14}H_{29}SO_5^-$	Anthropogenic, Diesel	no	0.14	0	7.00	-1.36	20, 22, 35, 45	–	–
311.01	$C_9H_{11}SO_{10}^-$	–	no	0.78	4	1.00	1.00	22	–	–
311.04	$C_{10}H_{15}SO_9^-$	–	no	0.60	3	-1.00	0.30	4, 35	Aqueous-phase •OH oxidation of α-pinene-derived organosulfate	29
311.08	$C_{11}H_{19}SO_8^-$	–	no	0.45	2	0.00	-0.27	4, 35	–	–
311.12	$C_{12}H_{23}SO_7^-$	–	no	0.33	1	7.00	-0.75	4	Heterogeneous •OH oxidation of dodecyl sulfate	40
313.02	$C_9H_{13}SO_{10}^-$	–	no	0.78	3	0.00	0.78	22	–	–
313.06	$C_{10}H_{17}SO_9^-$	–	no	0.60	2	7.00	0.10	4, 22	–	–
315.13	$C_{15}H_{23}SO_5^-$	–	no	0.13	4	2.43	-0.87	45	–	–
315.16	$C_{16}H_{27}SO_4^-$	–	no	0.06	3	2.33	-1.19	4	–	–
316.96	$C_{10}H_5SO_{10}^-$	–	no	0.70	8	2.60	1.50	20	–	–
317.11	$C_{14}H_{21}SO_6^-$	–	no	0.21	4	2.33	-0.64	4	–	–
317.14	$C_{15}H_{25}SO_5^-$	β-caryophyllene	no	0.13	3	2.20	-1.00	45	–	–
318.94	$C_9H_3SO_{11}^-$	–	no	0.89	8	2.56	2.11	20	–	–
319.09	$C_{13}H_{19}SO_7^-$	–	no	0.31	4	2.20	-0.38	4	–	–
319.16	$C_{15}H_{27}SO_5^-$	–	no	0.13	2	1.67	-1.13	35	–	–
319.19	$C_{16}H_{31}SO_4^-$	–	no	0.06	1	1.00	-1.44	4	–	–
321.10	$C_{13}H_{21}SO_7^-$	Diesel fuel	no	0.31	3	1.67	-0.54	4, 19, 35	–	–
321.14	$C_{14}H_{25}SO_6^-$	Anthropogenic, Sesquiterpene	no	0.21	2	1.00	-0.93	3, 4, 20, 22, 35	–	–
321.17	$C_{15}H_{29}SO_5^-$	Anthropogenic, Diesel fuel	no	0.13	1	-1.00	-1.27	7, 19, 20, 25, 35	–	–
321.21	$C_{16}H_{33}SO_4^-$	–*	no	0.06	0	0.00	-1.56	22	–	–
323.04	$C_{11}H_{15}SO_9^-$	–	no	0.55	4	1.67	0.27	4	–	–
323.08	$C_{12}H_{19}SO_8^-$	–	no	0.42	3	1.00	-0.25	4, 35	–	–
323.12	$C_{13}H_{23}SO_7^-$	–	no	0.31	2	-1.00	-0.69	3, 4, 35	–	–

323.15	$C_{14}H_{27}SO_6^-$	Sesquiterpene	no	0.21	1	0.00	-1.07	3, 4, 20, 22, 35, 45	–	–
323.19	$C_{15}H_{31}SO_5^-$	–	no	0.13	0	7.00	-1.40	35	–	–
325.06	$C_{11}H_{17}SO_9^-$	–	no	0.55	3	-1.00	0.09	4, 35	–	–
325.10	$C_{12}H_{21}SO_8^-$	Diesel fuel	no	0.42	2	0.00	-0.42	19	–	–
327.04	$C_{10}H_{15}SO_{10}^-$	–	no	0.70	3	0.00	0.50	4	Aqueous-phase $^{\bullet}OH$ oxidation of α-pinene-derived organosulfate	29
329.09	$C_{11}H_{21}SO_9^-$	α-Pinene	no	0.55	1	4.33	-0.27	**28**	–	–
331.07	$C_{10}H_{19}SO_{10}^-$	Isoprene	no	0.70	1	4.00	0.10	24	–	–
331.12	$C_{15}H_{23}SO_6^-$	–	no	0.20	4	2.33	-0.73	4	–	–
332.96	$C_{10}H_5SO_{11}^-$	–	no	0.80	8	2.56	1.70	20	–	–
333.09	$C_{10}H_{21}SO_{10}^-$	Isoprene, IEPOX	no	0.70	0	3.67	-0.10	11, 37, 48	–	–
333.10	$C_{14}H_{21}SO_7^-$	Sesquiterpene	no	0.29	4	2.20	-0.50	4, 22	–	–
333.14	$C_{15}H_{25}SO_6^-$	–	no	0.20	3	2.00	-0.87	4	–	–
333.17	$C_{16}H_{29}SO_5^-$	–	no	0.13	2	1.67	-1.19	35	–	–
334.97	$C_{10}H_7SO_{11}^-$	–	no	0.80	7	2.43	1.50	20	–	–
335.08	$C_{13}H_{19}SO_8^-$	–	no	0.38	4	2.00	-0.23	4, 35	–	–
335.12	$C_{14}H_{23}SO_7^-$	–	no	0.29	3	1.67	-0.64	3, 4, 35	–	–
335.15	$C_{15}H_{27}SO_6^-$	–	no	0.20	2	1.00	-1.00	35	–	–
335.19	$C_{16}H_{31}SO_5^-$	Anthropogenic	no	0.13	1	-1.00	-1.31	4, 20, 22, 35	–	–
337.06	$C_{12}H_{17}SO_9^-$	–	no	0.50	4	1.67	0.08	4	–	–
337.10	$C_{13}H_{21}SO_8^-$	–	no	0.38	3	1.00	-0.38	4, 35	–	–
337.13	$C_{14}H_{25}SO_7^-$	–	no	0.29	2	-1.00	-0.79	3, 4, 35	–	–
337.17	$C_{15}H_{29}SO_6^-$	–	no	0.20	1	0.00	-1.13	4, 35	–	–
337.20	$C_{16}H_{33}SO_5^-$	–	no	0.13	0	7.00	-1.44	22, 35	–	–
339.07	$C_{12}H_{19}SO_9^-$	Diesel fuel	no	0.50	3	-1.00	-0.08	4, 19, 35	–	–
341.05	$C_{11}H_{17}SO_{10}^-$	–	no	0.64	3	0.00	0.27	4	–	–
341.18	$C_{18}H_{29}SO_4^-$	–	no	0.06	4	2.50	-1.17	4	–	–
347.08	$C_{14}H_{19}SO_8^-$	–	no	0.36	5	2.33	-0.21	4	–	–

347.12	$C_{15}H_{23}SO_7^-$	Sesquiterpenes, β-Caryophyllene	no	0.27	4	2.20	-0.60	3, 4, 14	–	–
347.19	$C_{17}H_{31}SO_5^-$	–	no	0.12	2	1.67	-1.24	35	–	–
348.95	$C_{10}H_5SO_{12}^-$	–	no	0.90	8	2.50	1.90	20	–	–
349.10	$C_{14}H_{21}SO_8^-$	–	no	0.36	4	2.00	-0.36	4	–	–
349.13	$C_{15}H_{25}SO_7^-$	Sesquiterpene, α-Pinene, β-Caryophyllene	no	0.27	3	1.67	-0.73	3, 4, 22, 28	–	–
349.17	$C_{16}H_{29}SO_6^-$	–	no	0.19	2	1.00	-1.06	4, 35	–	–
349.20	$C_{17}H_{33}SO_5^-$	–	no	0.12	1	-1.00	-1.35	4, 35	–	–
349.24	$C_{18}H_{37}SO_4^-$	–*	no	0.06	0	0.00	-1.61	22	–	–
351.07	$C_{13}H_{19}SO_9^-$	–	no	0.46	4	1.67	-0.08	4	–	–
351.11	$C_{14}H_{23}SO_8^-$	Sesquiterpene, β-Caryophyllene, Diesel fuel	no	0.36	3	1.00	-0.50	3, 19, 35	–	–
351.15	$C_{15}H_{27}SO_7^-$	–	no	0.27	2	-1.00	-0.87	3, 4, 35	–	–
351.18	$C_{16}H_{31}SO_6^-$	–	no	0.19	1	0.00	-1.19	3, 4, 22, 35	–	–
353.05	$C_{12}H_{17}SO_{10}^-$	–	no	0.58	4	1.00	0.25	4	–	–
353.09	$C_{13}H_{21}SO_9^-$	–	no	0.46	3	-1.00	-0.23	4	–	–
353.13	$C_{14}H_{25}SO_8^-$	–	no	0.36	2	0.00	-0.64	4	–	–
355.07	$C_{12}H_{19}SO_{10}^-$	–	no	0.58	3	0.00	0.08	4	–	–
357.04	$C_{18}H_{13}SO_6^-$	–	no	0.17	12	2.82	-0.06	20	–	–
357.05	$C_{11}H_{17}SO_{11}^-$	–	no	0.73	3	7.00	0.45	20	–	–
358.99	$C_9H_{11}SO_{13}^-$	–	no	1.11	4	7.00	1.67	20	–	–
359.06	$C_{11}H_{19}SO_{11}^-$	–	no	0.73	2	4.33	0.27	20, 22	–	–
361.04	$C_{10}H_{17}SO_{12}^-$	–	no	0.90	2	4.00	0.70	20	–	–
361.10	$C_{15}H_{21}SO_8^-$	–	no	0.33	5	2.33	-0.33	4	–	–
361.20	$C_{18}H_{33}SO_5^-$	–	no	0.11	2	1.67	-1.28	35	–	–
363.11	$C_{15}H_{23}SO_8^-$	β-Caryophyllene	no	0.33	4	2.00	-0.47	3, 4	–	–
363.15	$C_{16}H_{27}SO_7^-$	–	no	0.25	3	1.67	-0.81	4	–	–
363.18	$C_{17}H_{31}SO_6^-$	–	no	0.18	2	1.00	-1.12	35	–	–
363.22	$C_{18}H_{35}SO_5^-$	Anthropogenic	no	0.11	1	-1.00	-1.39	20, 22, 35	–	–
365.09	$C_{14}H_{21}SO_9^-$	–	no	0.43	4	1.67	-0.21	4	–	–

365.13	$C_{15}H_{25}SO_8^-$	Diesel fuel	no	0.33	3	1.00	-0.60	3, 4, 19	–	–
365.16	$C_{16}H_{29}SO_7^-$	–	no	0.25	2	-1.00	-0.94	3, 4, 25, 35	–	–
365.20	$C_{17}H_{33}SO_6^-$	–	no	0.18	1	0.00	-1.24	22, 35	–	–
367.07	$C_{13}H_{19}SO_{10}^-$	–	no	0.54	4	1.00	0.08	4	–	–
367.11	$C_{14}H_{23}SO_9^-$	–	no	0.43	3	-1.00	-0.36	4	–	–
367.14	$C_{15}H_{27}SO_8^-$	–	no	0.33	2	0.00	-0.73	4	–	–
367.18	$C_{16}H_{31}SO_7^-$	–	no	0.25	1	7.00	-1.06	22	–	–
373.10	$C_{16}H_{21}SO_8^-$	Diesel fuel	no	0.31	6	2.50	-0.31	4, 19, 22	–	–
377.09	$C_{15}H_{21}SO_9^-$	–	no	0.40	5	2.20	-0.20	4	–	–
377.13	$C_{16}H_{25}SO_8^-$	–	no	0.31	4	2.00	-0.56	4	–	–
377.20	$C_{18}H_{33}SO_6^-$	–	no	0.17	2	1.00	-1.17	22, 35	–	–
377.24	$C_{19}H_{37}SO_5^-$	–	no	0.11	1	-1.00	-1.42	22, 35	–	–
377.27	$C_{20}H_{41}SO_4^-$	–*	no	0.05	0	0.00	-1.65	22	–	–
379.11	$C_{15}H_{23}SO_9^-$	–	no	0.40	4	1.67	-0.33	4	–	–
379.14	$C_{16}H_{27}SO_8^-$	–	no	0.31	3	1.00	-0.69	4	–	–
379.18	$C_{17}H_{31}SO_7^-$	–	no	0.24	2	-1.00	-1.00	35	–	–
379.22	$C_{18}H_{35}SO_6^-$	–	no	0.17	1	0.00	-1.28	22, 35	–	–
381.09	$C_{14}H_{21}SO_{10}^-$	–	no	0.50	4	1.00	-0.07	4	–	–
381.12	$C_{15}H_{25}SO_9^-$	–	no	0.40	3	-1.00	-0.47	4	–	–
381.16	$C_{16}H_{29}SO_8^-$	–	no	0.31	2	0.00	-0.81	4	–	–
388.05	$C_{15}H_{16}SO_{10}^-$	–	no	0.47	8	2.56	0.27	25	–	–
390.96	$C_{12}H_7SO_{13}^-$	–	no	0.83	9	2.56	1.58	20	–	–
391.22	$C_{19}H_{35}SO_6^-$	–	no	0.16	2	1.00	-1.21	35	–	–
391.25	$C_{20}H_{39}SO_5^-$	–	no	0.10	1	-1.00	-1.45	22, 35	–	–
393.17	$C_{21}H_{29}SO_5^-$	–	no	0.10	7	2.69	-0.90	22	–	–
393.19	$C_{18}H_{33}SO_7^-$	–	no	0.22	2	-1.00	-1.06	4, 22, 35	–	–
395.14	$C_{16}H_{27}SO_9^-$	–	no	0.38	3	-1.00	-0.56	4	–	–
397.12	$C_{15}H_{25}SO_{10}^-$	α-Pinene	no	0.47	3	0.00	-0.33	**28**	–	–
401.08	$C_{24}H_{17}SO_4^-$	–	no	0.04	16	2.88	-0.38	20	–	–
405.27	$C_{21}H_{41}SO_5^-$	Anthropogenic	no	0.10	1	-1.00	-1.48	20, 35	–	–
405.30	$C_{22}H_{45}SO_4^-$	–*	no	0.05	0	0.00	-1.68	22	–	–
407.19	$C_{22}H_{31}SO_5^-$	–	no	0.09	7	2.69	-0.95	22	–	–
409.19	$C_{18}H_{33}SO_8^-$	–	no	0.28	2	0.00	-0.94	4, 22	–	–

409.23	$C_{19}H_{37}SO_7^-$	–	no	0.21	1	7.00	-1.21	22	–	–
417.30	$C_{23}H_{45}SO_4^-$	–	no	0.04	1	1.00	-1.61	22	–	–
419.28	$C_{22}H_{43}SO_5^-$	–	no	0.09	1	-1.00	-1.50	35	–	–
419.32	$C_{23}H_{47}SO_4^-$	–*	no	0.04	0	0.00	-1.70	22	–	–
421.23	$C_{20}H_{37}SO_7^-$	–	no	0.20	2	-1.00	-1.15	22, 35	–	–
423.17	$C_{18}H_{31}SO_9^-$	–	no	0.33	3	-1.00	-0.72	4	–	–
425.22	$C_{19}H_{37}SO_8^-$	–	no	0.26	1	5.00	-1.11	22	–	–
431.17	$C_{20}H_{31}SO_8^-$	α-Pinene	no	0.25	5	2.33	-0.75	**28**	–	–
433.30	$C_{23}H_{45}SO_5^-$	–	no	0.09	1	-1.00	-1.52	35	–	–
433.34	$C_{24}H_{49}SO_4^-$	–*	no	0.04	0	0.00	-1.71	22	–	–
435.24	$C_{21}H_{39}SO_7^-$	–	no	0.19	2	-1.00	-1.19	22	–	–
437.26	$C_{21}H_{41}SO_7^-$	–	no	0.19	1	7.00	-1.29	22	–	–
439.09	$C_{23}H_{19}SO_7^-$	–	no	0.17	14	2.84	-0.22	20	–	–
447.35	$C_{25}H_{51}SO_4^-$	–*	no	0.04	0	0.00	-1.72	22	–	–
459.28	$C_{24}H_{43}SO_6^-$	–	no	0.13	3	2.00	-1.29	22	–	–
461.37	$C_{26}H_{53}SO_4^-$	–*	no	0.04	0	0.00	-1.73	22	–	–
463.31	$C_{24}H_{47}SO_6^-$	–	no	0.13	1	0.00	-1.46	35	–	–
465.10	$C_{25}H_{21}SO_7^-$	–	no	0.16	15	2.85	-0.28	20	–	–
466.11	$C_{25}H_{22}SO_7^-$	–	no	0.16	15	2.85	-0.32	25	–	–
485.22	$C_{24}H_{37}SO_8^-$	–	no	0.21	6	2.50	-0.88	35	–	–
489.10	$C_{27}H_{21}SO_7^-$	–	no	0.15	17	2.87	-0.26	20	–	–
503.32	$C_{30}H_{47}SO_4^-$	–	no	0.03	7	2.71	-1.30	22	–	–
507.30	$C_{25}H_{47}SO_8^-$	–	no	0.20	2	0.00	-1.24	22	–	–
569.26	$C_{25}H_{45}SO_{12}^-$	α-Pinene	no	0.36	3	5.00	-0.84	**28**	–	–
569.33	$C_{34}H_{49}SO_5^-$	–	no	0.06	10	2.79	-1.15	20	–	–
587.31	$C_{26}H_{51}SO_{12}^-$	Anthropogenic	no	0.35	1	3.67	-1.04	20	–	–
622.41	$C_{39}H_{58}SO_4^-$	–	no	0.03	11	2.81	-1.28	25	–	–
691.44	$C_{43}H_{63}SO_5^-$	–	no	0.05	12	2.83	-1.23	20	–	–

Table S1 was compiled through literature review using Web of Science database with keywords such as organosulfates, organosulfur compounds, aerosol, and field measurement. Additional searches were performed using individual OS molecular formulas and reported *m*/*z* values, and the reference list of relevant publications were also screened to ensure comprehensive coverage. The authors acknowledge that assigning suggested precursors based solely on molecular formulas, without supporting retention time (RT) information, has inherent limitations, particularly for OSs with multiple structural or stereoisomers. Consequently, the percentages shown in **Figure 1b**, which are derived from the compilation in **Table S1**, are therefore subject to uncertainty and should be interpreted with caution.

[*]represents saturated alkyl OSs, most likely of anthropogenic origin.

Section S2. Conversion between organic and inorganic sulfur upon chemical aging of OSs

Compared with freshly formed OSs, the chemical stability, reaction kinetics, mechanisms, and ultimate products of OSs formed at higher oxidation stages remain largely unexplored. Furthermore, laboratory evidence shows that the conversion of OS-bound sulfur to inorganic sulfate during chemical aging can be rapid. For example, heterogeneous $^{\bullet}OH$ oxidation efficiently drives inorganic sulfur release: 2-methyltetrol sulfates (2-MTS), among the most abundant atmospheric OSs, convert more than two-thirds of their organic sulfur to inorganic sulfate over approximately 15 days under typical $^{\bullet}OH$ concentrations, with inorganic sulfate formation increasing with $^{\bullet}OH$ exposure.[49] Aqueous-phase oxidation reactions under cloud-relevant conditions is effective: $^{\bullet}OH$ oxidation converts about 50% of OS-bound sulfur in α-pinene-derived OSs to inorganic sulfate within half a day at typical aqueous-phase $^{\bullet}OH$ levels, and inorganic sulfate continues to accumulate even after parent OS are fully oxidized.[29] Hydrolysis provides an additional non-oxidative mechanism for inorganic sulfate formation. The acid-catalyzed cleavage of $-OSO_3^-$ yields polyols and sulfuric acid, enabling complete conversion of OS-bound sulfur to inorganic sulfate.[50, 51] Altogether, we propose that OSs formed across different generations likely exist as reactive intermediate species that are continuously transformed upon aging, leading to a significant conversion of OS-bound sulfur to inorganic sulfate as a final product (**Scheme 1, main text**).

Experimental evidence shows that the extent of OS-bound sulfur conversion to inorganic sulfate generally increases as OSs become smaller and more oxygenated during transformation. This trend appears across OSs with different carbon numbers and reveals an inverse relationship between sulfur-conversion rate and molecular size (**Figure S1**).[16, 46, 49, 52-54] For example, upon heterogeneous $^{\bullet}OH$ oxidation, methyl sulfate (C_1) exhibits the highest sulfur conversion (74%),[52] followed by 2-MTS (C_5, 68%),[49] α-pinene-derived OSs (C_{10}, 14%),[46] and dodecyl sulfate (C_{12}, 2%).[40] This pattern suggests that smaller OSs (e.g., methyl sulfate) possess greater radical accessibility and more labile bonds, making them more susceptible to fragmentation and loss of the $-OSO_3^-$ group during oxidation. For larger and less oxygenated OSs (e.g., dodecyl sulfate), the early stages of $^{\bullet}OH$ oxidation typically yield more oxygenated OS products that retain the $-OSO_3^-$ group. These oxygenated intermediates may help explain some of the unclassified OS signals observed in ambient aerosols. At later stages, fragmentation becomes increasingly dominant, leading to enhanced formation of inorganic sulfate as more oxygenated compounds are tend to fragment more easily. This formation and accumulation of inorganic sulfate is consistently observed across diverse experimental systems,

including aqueous-phase oxidation and hydrolysis (**Figure S1**).[29] Collectively, these observations indicate that the formation and accumulation of inorganic sulfate from OS aging likely proceed through multigenerational reaction pathways, particularly for larger OSs.

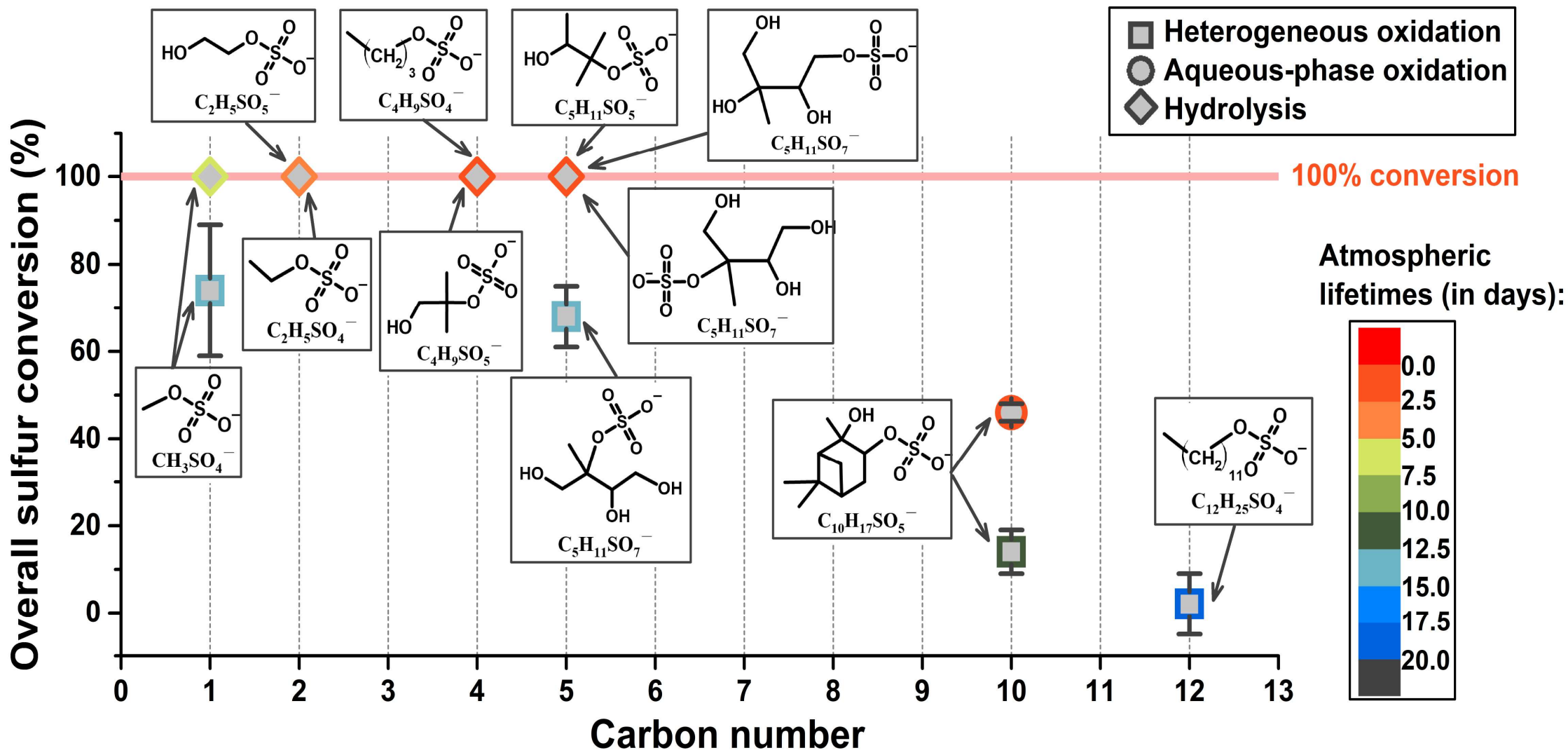

Figure S1. Summary of OSs subjected to multiple atmospheric aging processes and the resulting overall conversion of OS-bound sulfur to inorganic sulfate reported in previous studies.[29, 50, 55] Point color represents the corresponding atmospheric lifetimes (days). For each carbon number, only the shortest reported lifetime is shown and the dataset is provided in **Table S2**.

Table S2. Summary of OSs subjected to multiple atmospheric aging processes and the resulting overall conversion of sulfur from OSs to inorganic sulfate reported in previous studies.

Chemical structure	Chemical formula	Notes	Chemical aging pathways	Atmospheric lifetime	Detection methods for inorganic sulfate	Overall sulfur conversion	Ref.
	$CH_3O_4S^-$ (*m/z* 110.98)	Methyl sulfate	Heterogeneous ·OH oxidation	13.5 days[a]	Ion chromatography (IC)	74% ± 15%	52
	$C_{12}H_{25}O_4S^-$ (*m/z* 265.15)	Dodecyl sulfate	Heterogeneous ·OH oxidation	18.9 days[a]	Ion chromatography (IC)	2% ± 7%	40
	$C_{10}H_{17}O_5S^-$ (*m/z* 249.08)	*α*-pinene-derived OS (*α*pOS-249)	Heterogeneous ·OH oxidation	11.5 days[a]	Ion chromatography (IC)	14% ± 5%	46
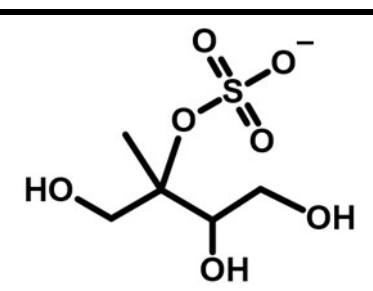	$C_5H_{11}O_7S^-$ (*m/z* 215.02)	2-methyltetrol sulfates (2-MTS)	Heterogeneous ·OH oxidation	14.4 days[a]	Ion chromatography (IC)	68% ± 7%	49
	$C_{10}H_{17}O_5S^-$ (*m/z* 249.08)	*α*-pinene-derived OS (*α*pOS-249)	Aqueous-phase ·OH oxidation	0.5 days[b]	Ion chromatography (IC)	46% ± 2%	29

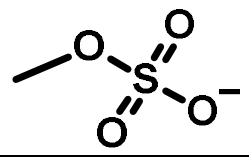	$CH_3O_4S^-$ (*m/z* 110.98)	Methyl sulfate	Gas-phase •OH oxidation	Not quantified	Quantum chemical calculations	Primary product	54
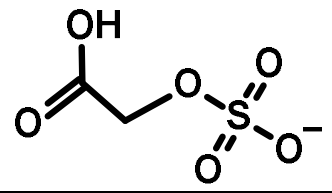	$C_2H_3O_6S^-$ (*m/z* 154.97)	Glycolic acid sulfate	Gas-phase •OH oxidation	Not quantified	Quantum chemical calculations	Primary product	54
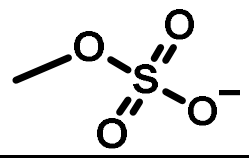	$CH_3O_4S^-$ (*m/z* 110.98)	Methyl sulfate	Aqueous-phase •OH oxidation	Not quantified	Quantum chemical calculations	Primary product	54
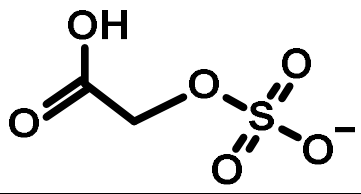	$C_2H_3O_6S^-$ (*m/z* 154.97)	Glycolic acid sulfate	Aqueous-phase •OH oxidation	Not quantified	Quantum chemical calculations	Primary product	54
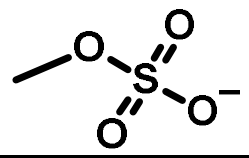	$CH_3O_4S^-$ (*m/z* 110.98)	Primary OS	Hydrolysis	5.0 days[c]	Nuclear magnetic resonance (NMR)	100% (end product)	50
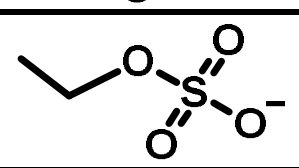	$C_2H_5O_4S^-$ (*m/z* 124.99)	Primary OS	Hydrolysis	4.2 days[c]	Nuclear magnetic resonance (NMR)	100% (end product)	50
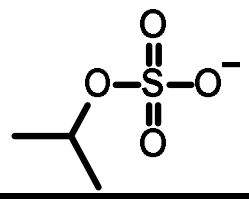	$C_3H_7O_4S^-$ (*m/z* 139.01)	Secondary OS	Hydrolysis	Not quantified	Nuclear magnetic resonance (NMR)	100% (end product)	50
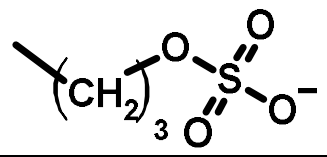	$C_4H_9O_4S^-$ (*m/z* 153.02)	Primary OS	Hydrolysis	10.8 days[c]	Nuclear magnetic resonance (NMR)	100% (end product)	50
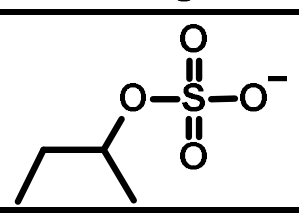	$C_4H_9O_4S^-$ (*m/z* 153.02)	Secondary OS	Hydrolysis	Not quantified	Nuclear magnetic resonance (NMR)	100% (end product)	50
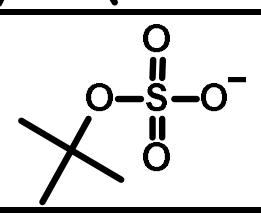	$C_4H_9O_4S^-$ (*m/z* 153.02)	Tertiary OS	Hydrolysis	Not quantified	Nuclear magnetic resonance (NMR)	100% (end product)	50
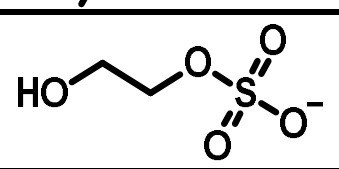	$C_2H_5O_5S^-$ (*m/z* 140.99)	Primary OS	Hydrolysis	3.0 days[d]	Nuclear magnetic resonance (NMR)	100% (end product)	50
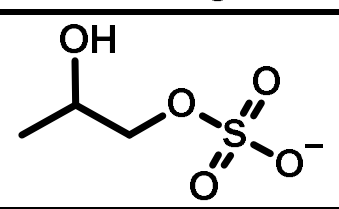	$C_3H_7O_5S^-$ (*m/z* 155.00)	Primary OS	Hydrolysis	Not quantified	Nuclear magnetic resonance (NMR)	100% (end product)	50
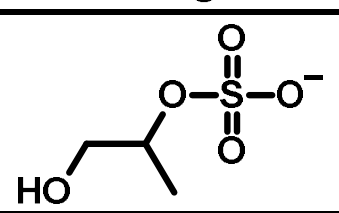	$C_3H_7O_5S^-$ (*m/z* 155.00)	Secondary OS	Hydrolysis	Not quantified	Nuclear magnetic resonance (NMR)	100% (end product)	50
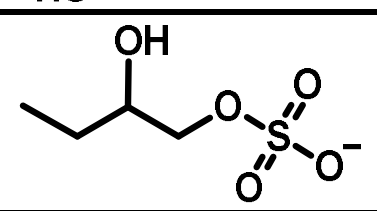	$C_4H_9O_5S^-$ (*m/z* 169.02)	Primary OS	Hydrolysis	Not quantified	Nuclear magnetic resonance (NMR)	100% (end product)	50
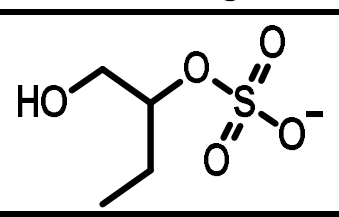	$C_4H_9O_5S^-$ (*m/z* 169.02)	Secondary OS	Hydrolysis	Not quantified	Nuclear magnetic resonance (NMR)	100% (end product)	50
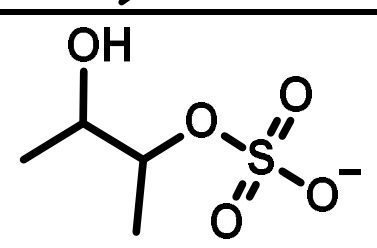	$C_4H_9O_5S^-$ (*m/z* 169.02)	Secondary OS	Hydrolysis	Not quantified	Nuclear magnetic resonance (NMR)	100% (end product)	50
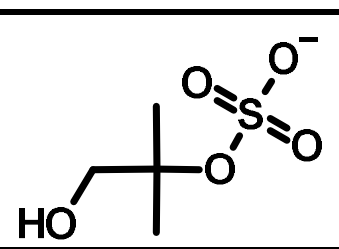	$C_4H_9O_5S^-$ (*m/z* 169.02)	Tertiary OS	Hydrolysis	0.1 days[d]	Nuclear magnetic resonance (NMR)	100% (end product)	50
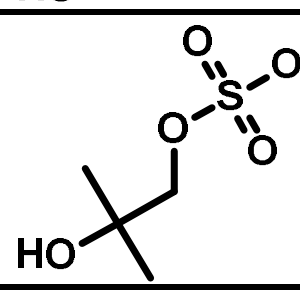	$C_4H_9O_5S^-$ (*m/z* 169.02)	Primary OS	Hydrolysis	Not quantified	Nuclear magnetic resonance (NMR)	100% (end product)	50

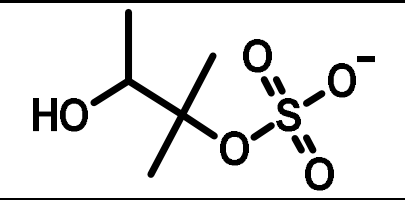	$C_5H_{11}O_5S^-$ (*m/z* 183.03)	Tertiary OS	Hydrolysis	1.3 days[d]	Nuclear magnetic resonance (NMR)	100% (end product)	50
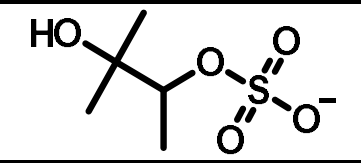	$C_5H_{11}O_5S^-$ (*m/z* 183.03)	Secondary OS	Hydrolysis	Not quantified	Nuclear magnetic resonance (NMR)	100% (end product)	50
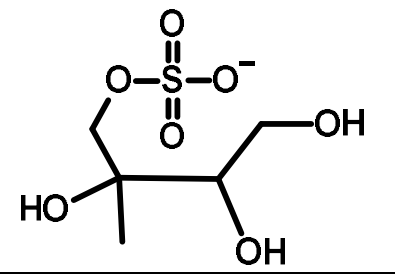	$C_5H_{11}O_7S^-$ (*m/z* 215.02)	Primary OS	Hydrolysis	Not quantified	Nuclear magnetic resonance (NMR)	100% (end product)	50
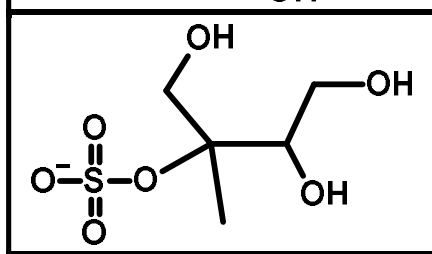	$C_5H_{11}O_7S^-$ (*m/z* 215.02)	Tertiary OS	Hydrolysis	19.2 days[d]	Nuclear magnetic resonance (NMR)	100% (end product)	50
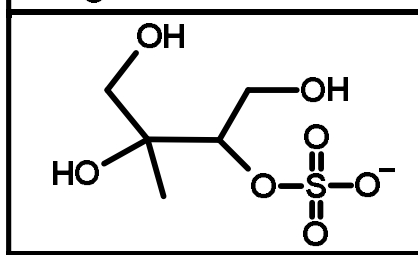	$C_5H_{11}O_7S^-$ (*m/z* 215.02)	Secondary OS	Hydrolysis	Not quantified	Nuclear magnetic resonance (NMR)	100% (end product)	50
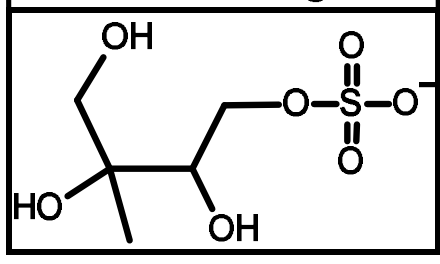	$C_5H_{11}O_7S^-$ (*m/z* 215.02)	Primary OS	Hydrolysis	>104.2 days[d]	Nuclear magnetic resonance (NMR)	100% (end product)	50

[a]Assuming an average ambient gas-phase $^{\bullet}$OH concentration, $[^{\bullet}OH]_{gas} = 1.5 \times 10^6$ molecules cm^{-3}.[52, 55]

[b]Assuming an average aqueous-phase $^{\bullet}$OH concentration, $[^{\bullet}OH]_{aqueous} = 1.0 \times 10^{-14}$ mol L^{-1}.[56]

[c]The acid-catalyzed mechanism hydrolysis lifetime (for 55 wt% D_2SO_4 solution) is given.

[d]For tertiary systems, the neutral mechanism hydrolysis lifetime is given. For primary and secondary systems, the acid-catalyzed mechanism hydrolysis lifetime (for 55 wt% D_2SO_4 solution) is given.

Section S3. Detailed mechanisms of OS-bound sulfur conversion to inorganic sulfate

Scheme S1 shows that OS-bound sulfur can be transformed to inorganic sulfate through both sulfur-centered radical and non-sulfur radical pathways. For instance, in sulfur-centered radical pathways, $^{\bullet}OH$-initiated oxidation of OSs begins with hydrogen abstraction from a C−H bond to form an alkyl radical ($R^{\bullet}$), which rapidly adds O_2 to produce a peroxyl radical ($RO_2^{\bullet}$). Subsequent $RO_2^{\bullet}$ self- or cross-reactions generate alkoxy radicals ($RO^{\bullet}$). When the $-OSO_3^-$ substituent is located at the α-position relative to the $RO^{\bullet}$ site, the resulting α−OSO_3^- alkoxy radical undergoes C–O(S) bond scission, yielding a sulfate radical anion ($SO_4^{\bullet-}$) and a non-sulfated organic product.[55] In aqueous media, $SO_4^{\bullet-}$ is efficiently converted to HSO_4^- and SO_4^{2-} (**Scheme S1a**).[53] Alternatively, the alkyl radical ($R^{\bullet}$) can undergo (C)O–S bond cleavage to form a sulfite radical anion ($SO_3^{\bullet-}$),[49, 54] which subsequently hydrolyzes or undergoes secondary reactions to produce sulfuric acid, HSO_4^-, and SO_4^{2-} (**Scheme S1b**).[54] Non-sulfur radical pathways also contribute to inorganic sulfate formation. Fragmentation of $RO^{\bullet}$ via β-scission or ester cleavage can release HSO_4^- (**Scheme S1c**).[54] Direct $^{\bullet}OH$ attack on the $-OSO_3^-$ group or adjacent α-carbon is generally not favored due to the strong electron-withdrawing nature of the $-OSO_3^-$, which suppresses α C–H abstraction.[29, 57] In addition, hydrolysis provides a non-oxidative route to inorganic sulfate formation, producing sulfuric acid and enabling complete conversion of OS-bound sulfur to inorganic sulfate (**Scheme S1d**).[50, 51] Concurrently, these pathways generate smaller, more volatile, non-sulfated organic fragments (e.g., aldehydes, ketones, alcohols, polyols, and small acids) alongside inorganic sulfate. These non-sulfated organics partition more readily to the gas phase, decreasing the particle-phase organic mass.[49] The combined effects of this volatility shift and sulfur mineralization potentially drive aerosol mass reduction and modify aerosol properties, such as hygroscopicity, oxidation state (O/C ratio).

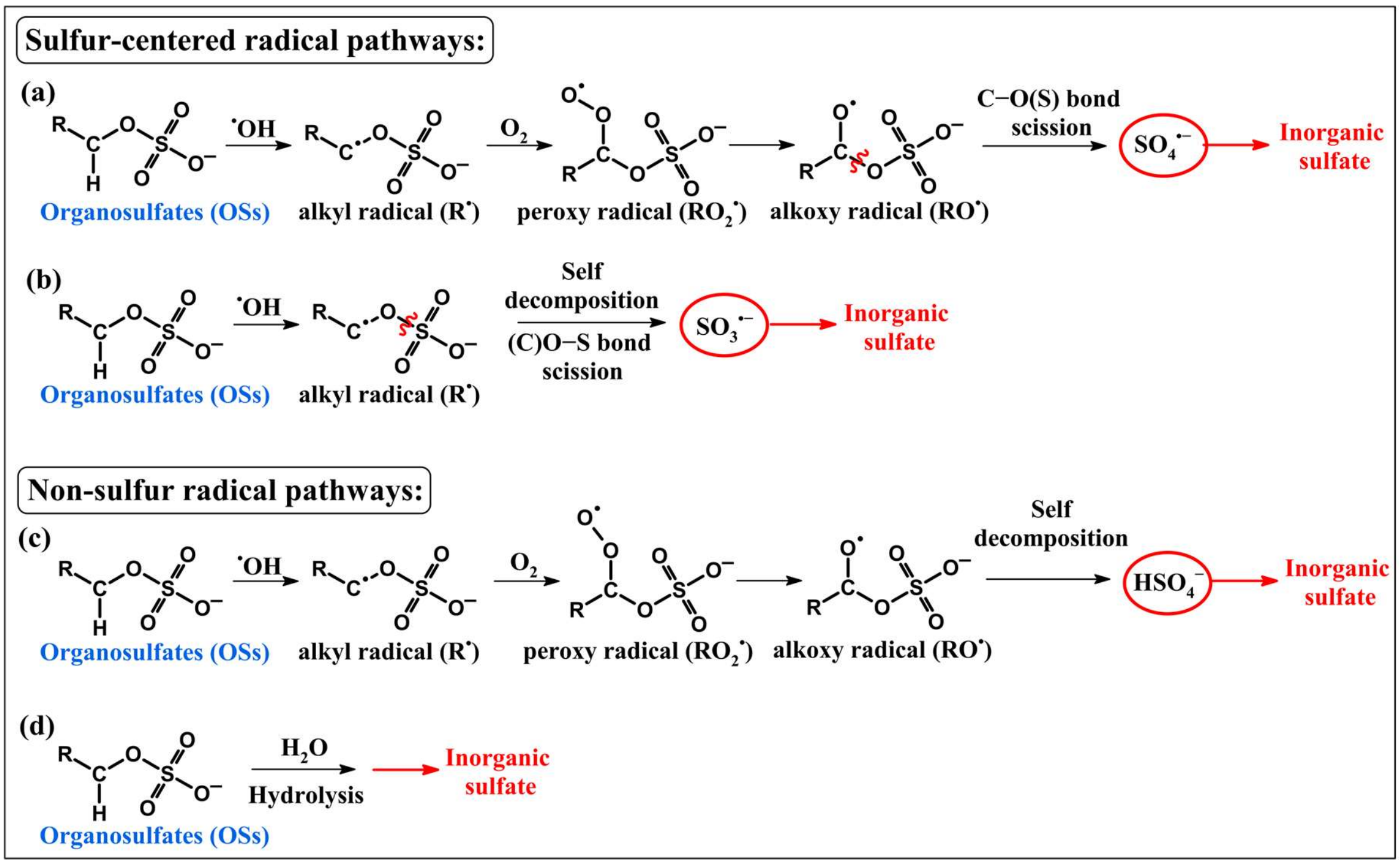

Scheme S1. Proposed pathways leading to inorganic sulfate formation from OSs, involving both sulfur-centered radical and non-sulfur radical pathways.

References

(1) Hettiyadura, A. P.; Jayarathne, T.; Baumann, K.; Goldstein, A. H.; de Gouw, J. A.; Koss, A.; Keutsch, F. N.; Skog, K.; Stone, E. A. Qualitative and quantitative analysis of atmospheric organosulfates in Centreville, Alabama. *Atmos. Chem. Phys.* **2017**, *17* (2), 1343-1359.

(2) Chen, Y. Z.; Dombek, T.; Hand, J.; Zhang, Z. F.; Gold, A.; Ault, A. P.; Levine, K. E.; Surratt, J. D. Seasonal Contribution of Isoprene-Derived Organosulfates to Total Water-Soluble Fine Particulate Organic Sulfur in the United States. *ACS Earth Space Chem.* **2021**, *5* (9), 2419-2432.

(3) Lin, P.; Yu, J. Z.; Engling, G.; Kalberer, M. Organosulfates in humic-like substance fraction isolated from aerosols at seven locations in East Asia: a study by ultra-high-resolution mass spectrometry. *Environ. Sci. Technol.* **2012**, *46* (24), 13118-13127.

(4) Kuang, B. Y.; Lin, P.; Hu, M.; Yu, J. Z. Aerosol size distribution characteristics of organosulfates in the Pearl River Delta region, China. *Atmos. Environ.* **2016**, *130*, 23-35.

(5) Liang, S.; Wang, Y.; Chen, H.; Chan, W.; Yu, J. Z. Accurate Quantification of Multifunctional $C_{2–3}$ Organosulfates in Atmospheric Aerosols Using Liquid Chromatography-Electrospray Ionization Mass Spectrometry: Overcoming Matrix Effects and Underestimation. *Environ. Sci. Technol.* **2025**, *59* (25), 12812-12821.

(6) Hettiyadura, A. P. S.; Al-Naiema, I. M.; Hughes, D. D.; Fang, T.; Stone, E. A. Organosulfates in Atlanta, Georgia: anthropogenic influences on biogenic secondary organic aerosol formation. *Atmos. Chem. Phys.* **2019**, *19* (5), 3191-3206.

(7) Huang, L.; Wang, Y.; Zhao, Y.; Hu, H.; Yang, Y.; Wang, Y.; Yu, J. Z.; Chen, T.; Cheng, Z.; Li, C. Biogenic and anthropogenic contributions to atmospheric organosulfates in a typical megacity in Eastern China. *J. Geophys. Res. Atmos.* **2023**, *128* (17), e2023JD038848.

(8) You, Y.-C.; Yang, T.; Xu, Y.; Gui, L.; Wang, Y.-C.; Yin, M.-J.; Xiao, H.; Xiao, H.-W.; Xiao, H.-Y. Novel Discoveries about the Potential Nonsecondary Origins of C_2–C_3 Organosulfates in Ambient Aerosol Particles. *Environ. Sci. Technol.* **2026**, *60* (8), 6298-6309.

(9) Zhu, Y.; Li, H.; Wei, J.; Ding, H.; Cai, D.; Zou, X.; Wang, G.; Wang, Q.; Ding, Z.; Liu, Z. Marine Modulation of the Formation Pathways and Sources of Organosulfates over the Coastal South China Sea. *Environ. Sci. Technol. Lett.* **2025**, *12* (6), 739-746.

(10) Glasius, M.; Hansen, A.; Claeys, M.; Henzing, J.; Jedynska, A.; Kasper-Giebl, A.; Kistler, M.; Kristensen, K.; Martinsson, J.; Maenhaut, W. Composition and sources of carbonaceous aerosols in Northern Europe during winter. *Atmos. Environ.* **2018**, *173*, 127-141.

(11) Rattanavaraha, W.; Chu, K.; Budisulistiorini, S. H.; Riva, M.; Lin, Y.-H.; Edgerton, E. S.; Baumann, K.; Shaw, S. L.; Guo, H.; King, L. Assessing the impact of anthropogenic pollution on isoprene-derived secondary organic aerosol formation in $PM_{2.5}$ collected from the Birmingham, Alabama, ground site during the 2013 Southern Oxidant and Aerosol Study. *Atmos. Chem. Phys.* **2016**, *16* (8), 4897-4914.

(12) Hansen, A. M. K.; Kristensen, K.; Nguyen, Q.; Zare, A.; Cozzi, F.; Nøjgaard, J. K.; Skov, H.; Brandt, J.; Christensen, J. H.; Ström, J. Organosulfates and organic acids in Arctic aerosols: speciation, annual variation and concentration levels. *Atmos. Chem. Phys.* **2014**, *14* (15), 7807-7823.

(13) Wang, Y.; Ma, Y.; Kuang, B.; Lin, P.; Liang, Y.; Huang, C.; Yu, J. Z. Abundance of organosulfates derived from biogenic volatile organic compounds: Seasonal and spatial contrasts at four sites in China. *Sci. Total. Environ.* **2022**, *806*, 151275.

(14) Stone, E. A.; Yang, L.; Liya, E. Y.; Rupakheti, M. Characterization of organosulfates in atmospheric aerosols at Four Asian locations. *Atmos. Environ.* **2012**, *47*, 323-329.

(15) Yang, Y.; Yan, C.; Yuan, R.; Liu, P.; Zhang, H.; Chen, H.; Zhu, Y.; Shen, H.; Wu, Y.; Xue, L. Liquid-phase reactions of aromatic organosulfates with OH radicals: kinetics, mechanisms, and environmental effects. *Atmos. Chem. Phys.* **2026**, *26* (11), 8211-8223.

(16) Kwong, K. C.; Chim, M. M.; Davies, J. F.; Wilson, K. R.; Chan, M. N. Importance of sulfate radical anion formation and chemistry in heterogeneous OH oxidation of sodium methyl sulfate, the smallest organosulfate. *Atmos. Chem. Phys.* **2018**, *18* (4), 2809-2820.

(17) Chen, Y. Z.; Zhang, Y.; Lambe, A. T.; Xu, R. S.; Lei, Z. Y.; Olson, N. E.; Zhang, Z. F.; Szalkowski, T.; Cui, T. Q.; Vizuete, W.; et al. Heterogeneous Hydroxyl Radical Oxidation of Isoprene-Epoxydiol-Derived Methyltetrol Sulfates: Plausible Formation Mechanisms of Previously Unexplained Organosulfates in Ambient Fine Aerosols. *Environ. Sci. Technol. Lett.* **2020**, *7* (7), 460-468.

(18) Hughes, D. D.; Stone, E. A. Organosulfates in the Midwestern United States: abundance, composition and stability. *Environ. Chem.* **2019**, *16* (5), 312-322.

(19) Wang, Y.; Hu, M.; Guo, S.; Wang, Y.; Zheng, J.; Yang, Y.; Zhu, W.; Tang, R.; Li, X.; Liu, Y. The secondary formation of organosulfates under interactions between biogenic emissions and anthropogenic pollutants in summer in Beijing. *Atmos. Chem. Phys.* **2018**, *18* (14), 10693-10713.

(20) Yang, T.; Xu, Y.; Ma, Y.-J.; Wang, Y.-C.; Yu, J. Z.; Sun, Q.-B.; Xiao, H.-W.; Xiao, H.-Y.; Liu, C.-Q. Field Evidence for Constraints of Nearly Dry and Weakly Acidic Aerosol

Conditions on the Formation of Organosulfates. *Environ. Sci. Technol. Lett.* **2024**, *11* (9), 981-987.
(21) Kristensen, K.; Bilde, M.; Aalto, P. P.; Petäjä, T.; Glasius, M. Denuder/filter sampling of organic acids and organosulfates at urban and boreal forest sites: Gas/particle distribution and possible sampling artifacts. *Atmos. Environ.* **2016**, *130*, 36-53.
(22) Qiu, Y.; Wang, J.; Qiu, T.; Li, J.; Bai, Y.; Liu, T.; Man, R.; Zong, T.; Fang, W.; Yang, J. Underestimation of anthropogenic organosulfates in atmospheric aerosols in urban regions. *Atmos. Chem. Phys.* **2026**, *26* (3), 2411-2423.
(23) Glasius, M.; Bering, M. S.; Yee, L. D.; de Sá, S. S.; Isaacman-VanWertz, G.; Wernis, R. A.; Barbosa, H. M.; Alexander, M. L.; Palm, B. B.; Hu, W. Organosulfates in aerosols downwind of an urban region in central Amazon. *Environ. Sci. Process. Impacts.* **2018**, *20* (11), 1546-1558.
(24) Ma, Y.; Xu, X.; Song, W.; Geng, F.; Wang, L. Seasonal and diurnal variations of particulate organosulfates in urban Shanghai, China. *Atmos. Environ.* **2014**, *85*, 152-160.
(25) Meade, L. E.; Riva, M.; Blomberg, M. Z.; Brock, A. K.; Qualters, E. M.; Siejack, R. A.; Ramakrishnan, K.; Surratt, J. D.; Kautzman, K. E. Seasonal variations of fine particulate organosulfates derived from biogenic and anthropogenic hydrocarbons in the mid-Atlantic United States. *Atmos. Environ.* **2016**, *145*, 405-414.
(26) Huang, R.-J.; Cao, J.; Chen, Y.; Yang, L.; Shen, J.; You, Q.; Wang, K.; Lin, C.; Xu, W.; Gao, B. Organosulfates in atmospheric aerosol: synthesis and quantitative analysis of $PM_{2.5}$ from Xi'an, northwestern China. *Atmos. Meas. Tech.* **2018**, *11* (6), 3447-3456.
(27) Wang, Y.; Zhang, Y.; Li, W.; Wu, G.; Qi, Y.; Li, S.; Zhu, W.; Yu, J. Z.; Yu, X.; Zhang, H.-H. Important roles and formation of atmospheric organosulfates in marine organic aerosols: influence of phytoplankton emissions and anthropogenic pollutants. *Environ. Sci. Technol.* **2023**, *57* (28), 10284-10294.
(28) Tiszenkel, L.; Pedersen, A. N.; Vasudevan-Geetha, V.; Hopf, M. C.; Glasius, M.; Elm, J.; Lee, S. H. Oxygenated organosulfates are an effective nucleation precursor in mixed biogenic and anthropogenic environments. *Geophys. Res. Lett.* **2026**, *53* (1), e2025GL117259.
(29) Lai, D. E.; Bai, Y. X.; Zhang, Z. J.; So, P.-K.; Li, Y. J.; Tse, Y.-L. S.; Yeung, Y.-Y.; Schaefer, T.; Herrmann, H.; Yu, J. Z. Rapid aqueous-phase oxidation of an α-pinene-derived organosulfate by hydroxyl radicals: a potential source of some unclassified oxygenated and small organosulfates in the atmosphere. *Atmos. Chem. Phys.* **2025**, *25* (19), 12569-12584.

(30) Gweme, D. T.; Styler, S. A. OH Radical Oxidation of Organosulfates in the Atmospheric Aqueous Phase. *J. Phys. Chem. A.* **2024**, *128* (43), 9462-9475.
(31) Le Breton, M.; Wang, Y.; Hallquist, Å. M.; Pathak, R. K.; Zheng, J.; Yang, Y.; Shang, D.; Glasius, M.; Bannan, T. J.; Liu, Q. Online gas-and particle-phase measurements of organosulfates, organosulfonates and nitrooxy organosulfates in Beijing utilizing a FIGAERO ToF-CIMS. *Atmos. Chem. Phys.* **2018**, *18* (14), 10355-10371.
(32) Olson, C. N.; Galloway, M. M.; Yu, G.; Hedman, C. J.; Lockett, M. R.; Yoon, T.; Stone, E. A.; Smith, L. M.; Keutsch, F. N. Hydroxycarboxylic acid-derived organosulfates: synthesis, stability, and quantification in ambient aerosol. *Environ. Sci. Technol.* **2011**, *45* (15), 6468-6474.
(33) Vogel, A. L.; Schneider, J.; Müller-Tautges, C.; Klimach, T.; Hoffmann, T. Aerosol chemistry resolved by mass spectrometry: Insights into particle growth after ambient new particle formation. *Environ. Sci. Technol.* **2016**, *50* (20), 10814-10822.
(34) Staudt, S.; Kundu, S.; Lehmler, H.-J.; He, X.; Cui, T.; Lin, Y.-H.; Kristensen, K.; Glasius, M.; Zhang, X.; Weber, R. J. Aromatic organosulfates in atmospheric aerosols: Synthesis, characterization, and abundance. *Atmos. Environ.* **2014**, *94*, 366-373.
(35) Tao, S.; Lu, X.; Levac, N.; Bateman, A. P.; Nguyen, T. B.; Bones, D. L.; Nizkorodov, S. A.; Laskin, J.; Laskin, A.; Yang, X. Molecular characterization of organosulfates in organic aerosols from Shanghai and Los Angeles urban areas by nanospray-desorption electrospray ionization high-resolution mass spectrometry. *Environ. Sci. Technol.* **2014**, *48* (18), 10993-11001.
(36) Hettiyadura, A.; Stone, E.; Kundu, S.; Baker, Z.; Geddes, E.; Richards, K.; Humphry, T. Determination of atmospheric organosulfates using HILIC chromatography with MS detection. *Atmos. Meas. Tech.* **2015**, *8* (6), 2347-2358.
(37) Cui, T.; Zeng, Z.; Dos Santos, E. O.; Zhang, Z.; Chen, Y.; Zhang, Y.; Rose, C. A.; Budisulistiorini, S. H.; Collins, L. B.; Bodnar, W. M. Development of a hydrophilic interaction liquid chromatography (HILIC) method for the chemical characterization of water-soluble isoprene epoxydiol (IEPOX)-derived secondary organic aerosol. *Environ. Sci. Process. Impacts.* **2018**, *20* (11), 1524-1536.
(38) He, Q.-F.; Ding, X.; Wang, X.-M.; Yu, J.-Z.; Fu, X.-X.; Liu, T.-Y.; Zhang, Z.; Xue, J.; Chen, D.-H.; Zhong, L.-J. Organosulfates from pinene and isoprene over the Pearl River Delta, South China: seasonal variation and implication in formation mechanisms. *Environ. Sci. Technol.* **2014**, *48* (16), 9236-9245.

(39) Riva, M.; Da Silva Barbosa, T.; Lin, Y.-H.; Stone, E. A.; Gold, A.; Surratt, J. D. Chemical characterization of organosulfates in secondary organic aerosol derived from the photooxidation of alkanes. *Atmos. Chem. Phys.* **2016**, *16* (17), 11001-11018.
(40) Ng, S. I. M.; Ng, K. H.; Yeung, P. W. F.; Xu, R.; So, P.-K.; Huang, Y.; Yu, J. Z.; Choi, C. K. K.; Tse, Y.-L. S.; Chan, M. N. Chemical Transformation of a Long-Chain Alkyl Organosulfate via Heterogeneous OH Oxidation: A Case Study of Sodium Dodecyl Sulfate. *Environ. Sci. Atmos.* **2022**, *2* (5): 1060–1075.
(41) Gómez-González, Y.; Wang, W.; Vermeylen, R.; Chi, X.; Neirynck, J.; Janssens, I.; Maenhaut, W.; Claeys, M. Chemical characterisation of atmospheric aerosols during a 2007 summer field campaign at Brasschaat, Belgium: sources and source processes of biogenic secondary organic aerosol. *Atmos. Chem. Phys.* **2012**, *12* (1), 125-138.
(42) Kristensen, K.; Glasius, M. Organosulfates and oxidation products from biogenic hydrocarbons in fine aerosols from a forest in North West Europe during spring. *Atmos. Environ.* **2011**, *45* (27), 4546-4556.
(43) Lin, Y.-H.; Knipping, E.; Edgerton, E.; Shaw, S.; Surratt, J. Investigating the influences of SO_2 and NH_3 levels on isoprene-derived secondary organic aerosol formation using conditional sampling approaches. *Atmos. Chem. Phys.* **2013**, *13* (16), 8457-8470.
(44) Riva, M.; Tomaz, S.; Cui, T.; Lin, Y.-H.; Perraudin, E.; Gold, A.; Stone, E. A.; Villenave, E.; Surratt, J. D. Evidence for an unrecognized secondary anthropogenic source of organosulfates and sulfonates: gas-phase oxidation of polycyclic aromatic hydrocarbons in the presence of sulfate aerosol. *Environ. Sci. Technol.* **2015**, *49* (11), 6654-6664.
(45) Brüggemann, M.; van Pinxteren, D.; Wang, Y.; Yu, J. Z.; Herrmann, H. Quantification of known and unknown terpenoid organosulfates in PM_{10} using untargeted LC–HRMS/MS: contrasting summertime rural Germany and the North China Plain. *Environ. Chem.* **2019**, *16* (5), 333-346.
(46) Xu, R. S.; Ng, S. I. M.; Chow, W. S.; Wong, Y. K.; Wang, Y. C.; Lai, D.; Yao, Z. P.; So, P. K.; Yu, J. Z.; Chan, M. N. Chemical transformation of *α*-pinene-derived organosulfate via heterogeneous OH oxidation: implications for sources and environmental fates of atmospheric organosulfates. *Atmos. Chem. Phys.* **2022**, *22* (8), 5685-5700.
(47) Wang, Y.; Ren, J.; Huang, X. H.; Tong, R.; Yu, J. Z. Synthesis of four monoterpene-derived organosulfates and their quantification in atmospheric aerosol samples. *Environ. Sci. Technol.* **2017**, *51* (12), 6791-6801.

(48) Nguyen, Q.; Christensen, M. K.; Cozzi, F.; Zare, A.; Hansen, A. M. K.; Kristensen, K.; Tulinius, T. E.; Madsen, H.; Christensen, J. H.; Brandt, J. Understanding the anthropogenic influence on formation of biogenic secondary organic aerosols in Denmark via analysis of organosulfates and related oxidation products. *Atmos. Chem. Phys.* **2014**, *14* (17), 8961-8981.
(49) Xu, R. S.; Chen, Y.; Ng, S. I. M.; Zhang, Z.; Gold, A.; Turpin, B. J.; Ault, A. P.; Surratt, J. D.; Chan, M. N. Formation of Inorganic Sulfate and Volatile Nonsulfated Products from Heterogeneous Hydroxyl Radical Oxidation of 2-Methyltetrol Sulfate Aerosols: Mechanisms and Atmospheric Implications. *Environ. Sci. Technol. Lett.* **2024**, *11* (9), 968-974.
(50) Hu, K. S.; Darer, A. I.; Elrod, M. J. Thermodynamics and kinetics of the hydrolysis of atmospherically relevant organonitrates and organosulfates. *Atmos. Chem. Phys.* **2011**, *11* (16), 8307-8320.
(51) Darer, A. I.; Cole-Filipiak, N. C.; O'Connor, A. E.; Elrod, M. J. Formation and stability of atmospherically relevant isoprene-derived organosulfates and organonitrates. *Environ. Sci. Technol.* **2011**, *45* (5), 1895-1902.
(52) Xu, R. S.; Ge, Y.; Kwong, K. C.; Poon, H. Y.; Wilson, K. R.; Yu, J. Z.; Chan, M. N. Inorganic Sulfur Species Formed upon Heterogeneous OH Oxidation of Organosulfates: A Case Study of Methyl Sulfate. *ACS Earth Space Chem.* **2020**, *4* (11), 2041-2049.
(53) Lai, D. E.; Wong, Y. K.; Xu, R. S.; Xing, S. N.; Ng, S. I. M.; Kong, L.; Yu, J. Z.; Huang, D. D.; Chan, M. N. Significant Conversion of Organic Sulfur from Hydroxymethanesulfonate to Inorganic Sulfate and Peroxydisulfate Ions upon Heterogeneous OH Oxidation. *Environ. Sci. Technol. Lett.* **2023**, *10* (9), 773-778.
(54) Tsona Tchinda, N.; Lv, X.; Tasheh, S. N.; Ghogomu, J. N.; Du, L. Atmospheric fate of organosulfates through gas-phase and aqueous-phase reactions with hydroxyl radicals: implications for inorganic sulfate formation. *Atmos. Chem. Phys.* **2025**, *25* (15), 8575-8590.
(55) Ng, S. I. M.; Chan, M. N. Beyond the formation: unveiling the atmospheric transformation of organosulfates via heterogeneous OH oxidation. *Chem. Commun.* **2023**, *59* (94), 13919-13938.
(56) González-Sánchez, J. M.; Brun, N.; Wu, J.; Morin, J.; Temime-Roussel, B.; Ravier, S.; Mouchel-Vallon, C.; Clément, J.-L.; Monod, A. On the importance of atmospheric loss of organic nitrates by aqueous-phase $^{\bullet}$OH oxidation. *Atmos. Chem. Phys.* **2021**, *21* (6), 4915-4937.
(57) Lai, D. E.; Schaefer, T.; Zhang, Y. M.; Li, Y. J.; Xing, S. N.; Herrmann, H.; Chan, M. N. Deactivating Effect of Hydroxyl Radicals Reactivity by Sulfate and Sulfite Functional Groups

in Aqueous Phase ─ Atmospheric Implications for Small Organosulfur Compounds. *ACS ES&T Air*. **2024**, 1, 7, 678–689.
